# Heritable transgenic schistosomes as a living platform for SARS-CoV-2 neutralizing antibody secretion

**DOI:** 10.1038/s41467-026-76465-9

**Published:** 2026-08-26

**Authors:** Wannaporn Ittiprasert, Michael J. Smout, Victoria H. Mann, Matthew Moyle, Sean M. Kinahan, Daniel N. Ackerman, Danielle N. Rivera, Joshua L. Santarpia, Eric C. Carnes, Margaret M. Mentink-Kane, Marina Reis Costa, Cornelis H. Hokke, Meta Roestenberg, Maria Elena Bottazzi, Bethany K. Bracken, Bruce A. Rosa, Sergej Djuranovic, Darren A. Pickering, Paul R. Giacomin, Daniel Watterson, Naphak Modhiran, Max F. Moescheid, Christoph G. Grevelding, Makedonka Mitreva, Alex Loukas, Paul J. Brindley

**Affiliations:** 1 1Department of Microbiology, Immunology & Tropical Medicine, School of Medicine & Health Sciences, George Washington University, Washington, D.C USA; 2 Australian Institute of Tropical Health and Medicine, James Cook University, Cairns, QLD Australia; 3 College of Medicine and Dentistry, James Cook University, Cairns, Australia; 4 Department of Pathology, Microbiology and Immunology, College of Medicine, and Global Center for Health Security University of Nebraska Medical Center, Omaha, NE USA; 5 Biological Defense Directorate, National Strategic Research Institute, University of Nebraska Medical Center, Omaha, NE USA; 6 Department of Environmental, Agricultural, and Occupational Health, College of Public Health, University of Nebraska Medical Center, Omaha, NE USA; 7 Schistosomiasis Resource Center, Biomedical Research Institute, Rockville, MD USA; 8 Leiden University Medical Centre, Leiden, The Netherlands; 9 National School of Tropical Medicine, College of Medicine, Houston, TX USA; 10 Department of Pediatrics, Division of Pediatric Tropical Medicine, and Texas Children’s Hospital Center for Vaccine Development, Baylor College of Medicine, Houston, TX USA; 11 Charles River Analytics, Cambridge, MA UK; 12 Department of Medicine, Washington University of St. Louis School of Medicine, St. Louis, MO USA; 13 Department of Cell Biology and Physiology, Washington University of St. Louis School of Medicine, St. Louis, MO USA; 14 Department of Molecular Biology, Cell Biology and Biochemistry, Division of Biology and Medicine, Brown University, Providence, RI USA; 15 University of Montenegro, Institute for Interdisciplinary and Multidisciplinary Studies, Podgorica, Montenegro; 16 School of Chemistry and Molecular Biosciences, University of Queensland, St. Lucia, QLD Australia; 17 Australian Infectious Diseases Research Centre, Brisbane, QLD Australia; 18 Institute of Parasitology, Biomedical Research Center Seltersberg, Justus Liebig University, Giessen, Germany; 19 Department of Genetics, Washington University School of Medicine, St. Louis, MO USA; 20 McDonnell Genome Institute, Washington University of St. Louis, St. Louis, MO USA

**Keywords:** Parasite genetics, Parasitic infection, CRISPR-Cas9 genome editing, Mutagenesis, Immunization

## Abstract

We report the generation and propagation of not only the first heritable transgenic schistosome line but also a line that secretes a functional therapeutic protein in vivo. Using multiplexed CRISPR/Cas-mediated homology-directed knock-in targeted to a predicted genomic safe-harbor, we inserted a VHH–IgG1 Fc (termed C5-Fc) transgene into *Schistosoma mansoni* eggs. Single-miracidium infections of *Biomphalaria glabrata* yielded parental P0 lines; serial passage through snail and mouse hosts produced an F2 cohort in which all parasites carried the C5-Fc transgene and secreted C5-Fc into the murine venous circulation. Molecular assays confirmed chromosomal insertion, germline transmission and systemic secretion. Sera from mice harboring C5-Fc transgenic worms neutralized SARS-CoV-2 in vitro with potent activity consistent with the expected ACE2-binding blockade by the C5 variable domain of heavy-chain-only antibody (VHH). These results demonstrate (i) stable, heritable transgenesis of a platyhelminth, (ii) delivery of a biologically active antibody fragment by a live helminth in a mammalian host, and (iii) feasibility of using transgenic schistosomes as sustained, single-dose protein delivery platforms. This technology and delivery system enable new experimental approaches for schistosome biology and motivate exploration of living-foundry therapeutics.

## Abstract (web summary)

A heritable CRISPR knock-in in *Schistosoma mansoni*generated a transgenic line that secreted a functional anti–SARS-CoV-2 antibody into mouse circulation. Virus neutralization by infected mouse sera demonstrated a worm-based living biofoundry for sustained, in vivo biologic delivery.

## Introduction

To date, heritably transgenic lines of schistosomes, or of any parasitic platyhelminth – clade Neodermata within phylum Platyhelminthes, have not been described. If such lines were available, progress in deciphering the biology and pathophysiology of these major neglected tropical disease (NTD) pathogens would be hastened, for example on fundamental questions such as what controls host range and specificity of the schistosome species, how do schistosomes locate their predilection site within the human host, which factors are decisive for bidirectional female-male interaction, and which genes encode target proteins of therapeutic value. As with other species, the ability to manipulate the schistosome would represent a step-change for the field and enable deep investigation of the (patho)biological nature of this pathogen. Transgenic lines of schistosomes also would be useful for investigating the value of helminth therapy for autoimmune and metabolic diseases^1–3^. The recent development of human challenge models of single-sex schistosome infections^4,5^ paves the way for clinical assessment of transgenic schistosomes as living foundries for synthetic biology applications^6^. Pertinent also are recent reports that exposure to lung-migrating helminths, including schistosomes, protect against Severe Acute Respiratory Syndrome Coronavirus 2 (SARS-CoV-2) infection through macrophage-dependent T cell activation and remodeling of the lung environment^7,8^. Tractable, routine derivation of transgenic lines of schistosomes, and indeed other helminths that are used therapeutically in human challenge models^9^, would be unarguably welcome.

Deploying Clustered Regularly Interspaced Short Palindromic Repeats/CRISPR-associated (proteins) (CRISPR/Cas)-driven functional genomics including programmed knock out (KO) and knock-in (KI) mutagenesis for schistosomes was first reported in 2019, in research from our laboratory^10^. However, heritable transgenesis via insertion of a transgene into the germ line had not been achieved, reflecting multiple obstacles: a complex life cycle that cannot be completed in vitro, difficulties with transfection in a multicellular organism, limited germ-line access, lack of chromosomal safe-harbor sites, diploidy and dioecy, and sparse knowledge of secretion. We now report this advance, enabled by a combined suite of technical and conceptual innovations: 1) targeting transgene insertion to a predicted genome safe harbor site^11,12^; 2) deploying several overlapping guide RNAs to enhance the likelihood of RNA-guided chromosomal double stranded break (DSB) and homology directed repair (HDR) by transgene insertion^13–15^; and 3) by transfecting the immature egg stage of the schistosome^16^. Our goal was vertical transgene transmission via natural infection of the snail by the (transgenic) miracidium larval stage, and propagation of the transgenic schistosome line.

**Fig. 1:**
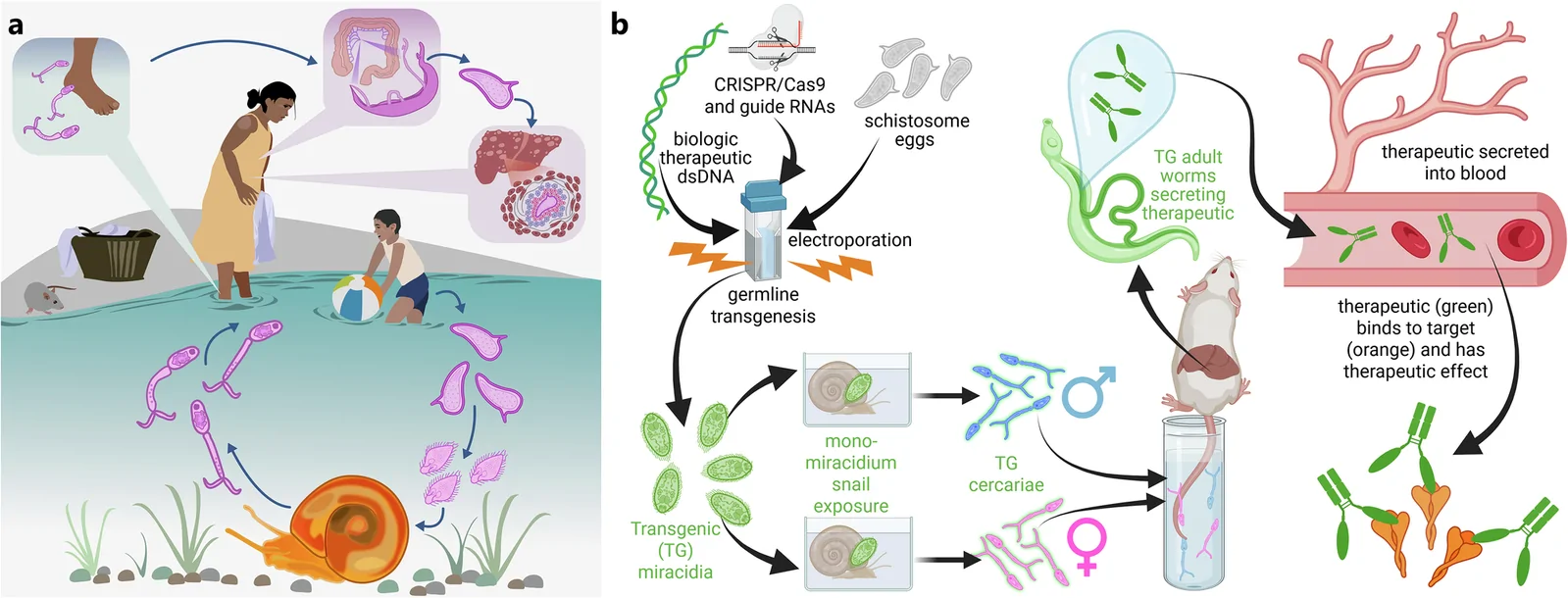
Schistosoma mansoni life cycle and strategy for generating heritable transgenic flukes. **a** Life cycle highlighting human infection. Cercariae, released from *Biomphalaria* snails, penetrate skin and transform into schistosomula. These migrate via circulation and lungs to the hepatic portal system, where the adult female and male schistosomes pair and females deposit eggs. Eggs traverse the gut for fecal excretion or lodge in tissues, causing hepato-intestinal disease. Eggs hatch in water to release miracidia, which infect snails and develop asexually into sporocysts that produce cercariae. **b** Transgenesis workflow in the laboratory, using the mouse as a surrogate for the human definitive host^47^. Immature eggs (containing the schistosome zygote) were transfected with CRISPR components and transgenes^12,16^. Single-miracidium infections of *B. glabrata* (NMRI strain)^40,87^ yield clonal, single-sex cercariae which infect mice, pair, and propagate transgenic lines. Transgenic schistosomes delivering a therapeutic encoded by the transgene represents a major goal of this endeavor^88^. Panel 1b created in BioRender. Smout, M. (2026) https://BioRender.com/2e178ah.

Derivation of a heritably transgenic line of *S. mansoni* represents transformative progress in tropical medicine. The line was established using CRISPR-based knock-in of an antibody-encoding transgene into the genome of the egg stage of *S. mansoni*. A parental female P0 and a parental male P0 generation were established by single miracidium exposure of the intermediate host, the aquatic gastropod *Biomphalaria glabrata*. Mice were infected with these P0 generation female and male line cercariae. Eggs subsequently recovered from the mice represented the F1 generation of the transgenic line, which we termed the “C5-Fc line”. In turn, snails were infected with miracidia hatched from the eggs. In this way, the C5-Fc line was propagated through to the F2 generation (Fig. 1). The transgene encodes an IgG1 Fc-fusion of C5, a variable domain of heavy-chain-only antibody (VHH)^17^, which targets the angiotensin converting enzyme 2 (ACE2) receptor binding site of the spike protein of SARS-CoV-2^18^. The C5-Fc line of schistosomes secreted the VHH into the blood of mice infected with these worms, and sera from these mice potently neutralized the SARS-CoV-2 virion.

## Results

### Virus neutralization by recombinant camelid antibodies

Amino acid sequences of three VHHs targeting the SARS-CoV-2 spike protein were obtained from the literature – VHH-C5^18^ and VHH-E and VHH-V^19^ along with a variant of VHH-C5 that was fused to the human IgG1 Fc domain, which we termed VHH-C5-Fc (Supplementary Fig. 1). All four VHHs expressed at high concentrations in HEK293F cells and were isolated in recombinant forms to >95% purity with single step, immobilized metal ion affinity chromatography (IMAC) (Supplementary Fig. 2a). Recombinant VHHs were assayed for virus neutralizing capacity using two independent assays: 1) an immunofluorescence-visualized pseudotyped lentiviral plaque assay^20^, and 2) by the SARS-CoV-2 plaque reduction neutralization test (PRNT)^21^. For the pseudotyped lentiviral plaque assay, neutralization was benchmarked against the Human SARS-CoV-2 Convalescent Serology Standard from the National Institute for Biological Standards and Control (NIBSC), NIBSC 20/130^22^. All VHHs neutralized pseudotyped lentivirus in the low microgram (VHH-E, VHH-V) to nanogram per milliliter ranges (VHH-C5, VHH-C5-Fc) VHH-C5-Fc was more potent than VHH-C5 with IC_50_ values of 5 ng/ml and 273 ng/ml, respectively (Supplementary Fig. 2b).

**Fig. 2:**
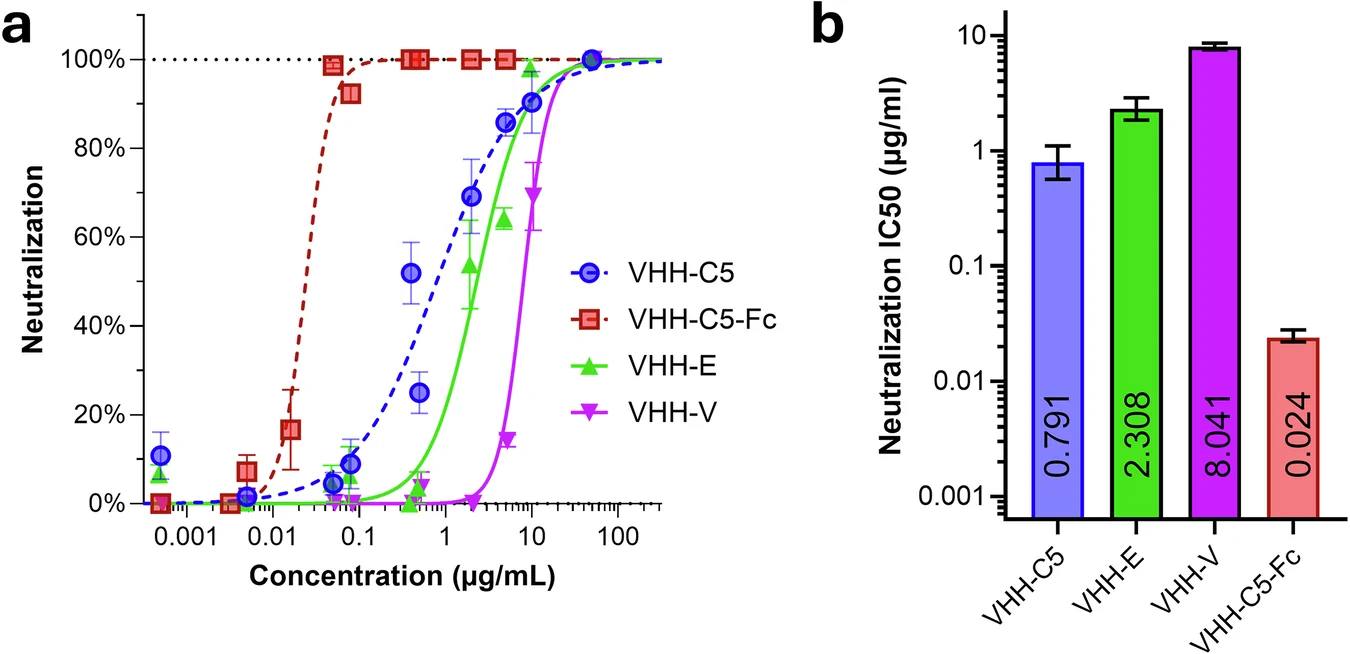
Neutralization of SARS-CoV-2 by recombinant VHHs expressed in HEK293F cells. **a** Dose-dependent neutralization measured by plaque reduction neutralization test. Percent reduction in plaque counts (relative to virus-only control), with each data point the mean of 3 technical replicates ± SEM bars, plotted against VHH concentration. **b** IC₅₀ values calculated from panel A using log (inhibitor) vs normalized response with variable Hill slope; bars represent best-fit values for IC₅₀ with 95% confidence interval error bars. Source data are provided as a Source Data file.

After confirming the neutralizing activity of the VHHs against pseudotyped lentivirus (an assay surrogate for SARS-CoV-2), we tested their neutralizing capacity directly against an isolate, SARS-CoV-2 USA-WA1/2020^23^, by the PRNT^21^. Neutralization of SARS-CoV-2 in this assay was benchmarked against the Human SARS-CoV-2 Serology Standard from the Frederick National Laboratory for Cancer Research (https://frederick.cancer.gov/resources/universal-standards-reference-materials/human-sars-cov-2-serology-standard)^22,24^; referred to here as FNLCR serum. Dose-dependent neutralization was obtained with all VHHs (Fig. 2a) and as also seen in the pseudotyped lentivirus assay, VHH-C5 and VHH-C5-Fc were the more potent of these VHHs, with IC_50_ values of 791 ng/ml and 24 ng/ml, respectively (Fig. 2b).

**Fig. 3:**
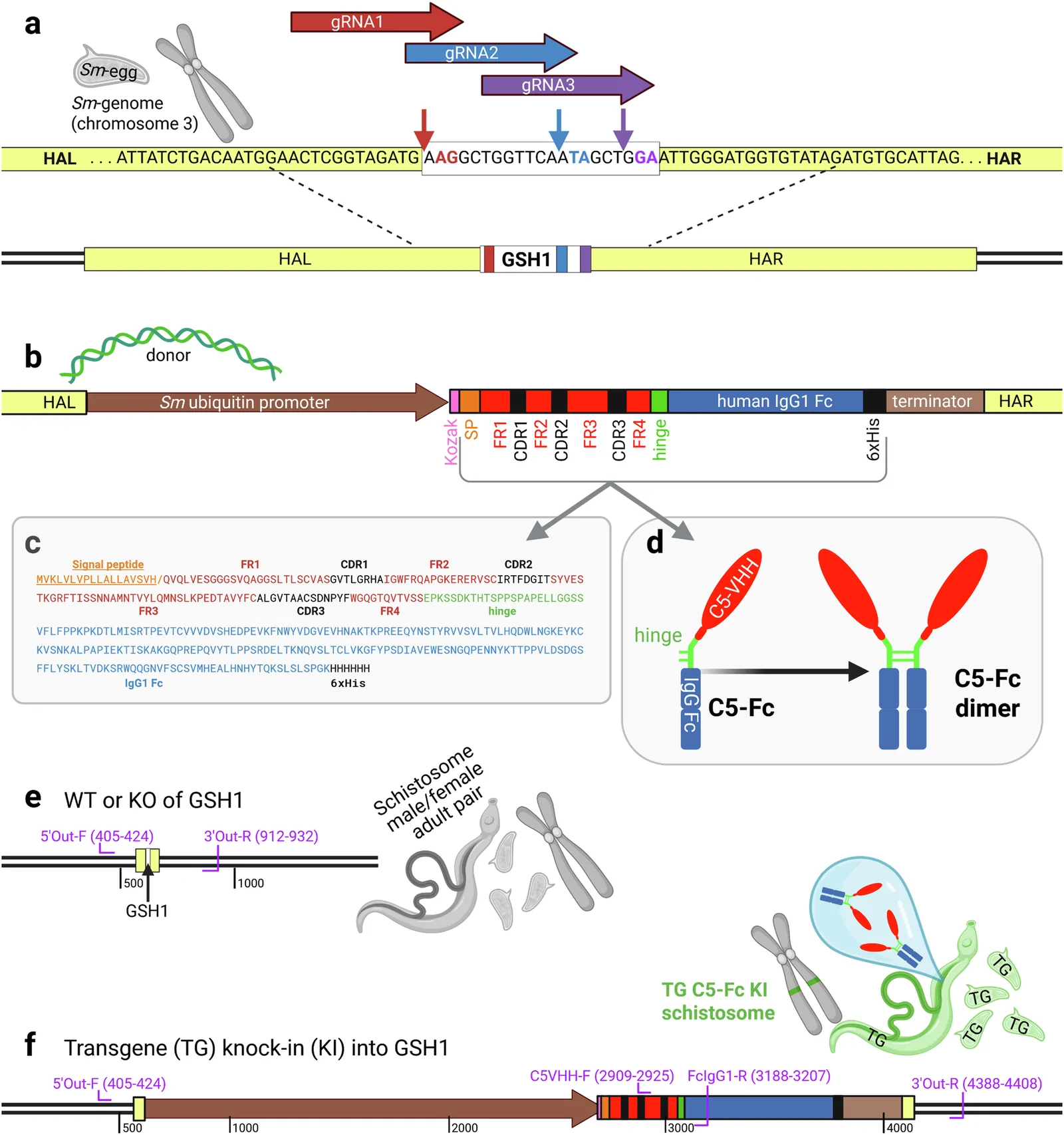
Assembly of transgene encoding anti-SARS-CoV-2 VHH. **a** Schematic depiction of the programmed CRISPR target site in *S. mansoni* Genome Safe Harbor 1 (GSH1), located on chromosome 3^12^. RNA-programmed CRISPR cleavage sites for three overlapping guide RNAs (gRNA1-3; red, blue, and purple arrows for programmed targets 1, 2 and 3, respectively). Yellow highlights/boxes indicate left and right homology arms (HAL, HAR) used to target the donor transgene. Genome sequences flanking the HAL/R are represented by two black parallel lines. **b** The C5-Fc coding regions designed for receptor binding domain of the spike protein of SARS-CoV-2 as three hypervariable domains or complementary determining regions (CDRs, Kabat–Chothia assignment)^89^ linked together with framework regions (FRs - red) with upstream Kozak motif (pink) and signal peptide (SP - orange) from the hookworm *Ancylostoma caninum* secreted protein ASP-2 (GenBank AF089728.1). The VHH coding regions is followed downstream by a hinge (green), IgG-Fc region (blue), and 6x histidine tag (black). The coding region is flanked by the schistosome ubiquitin gene promoter and terminator (brown) and further flanked by the HAL/HAR sequences (yellow). The transgene has been assigned GenBank accession PX933511. **c** Amino acid residues of the schistosome expressed C5-Fc, including its signal sequence. **d** Protein cartoon depiction of the expressed C5-Fc anti-SARS-CoV-2 VHH nanobody (red), hinge (green), IgG1 Fc (blue). The VHH-IgG1 fusion protein dimerizes because of the natural homodimerization of the IgG1 Fc domain. **e** Schematic depiction of unmodified wild-type/GSH1 or knock-out (KO) with grey schistosome adults, eggs, and chromosome. **f** Integrated transgene with green transgenic (TG) schistosomes and TG chromosome with green band representing donor gene insertion. Primer pairs (purple) specific for the GSH1 locus – 5’Out-F and 5’Out-R, expected amplicon of 4004 bp, and second, the TG – C5FcVHH-F and IgG1Fc-R, expected amplicon of 302 bp, designed to confirm transgene knock-in (KI) (primers sequences, Supplementary Table 1). Created in BioRender. Smout, M. (2026) https://BioRender.com/c267667.

Based on the IC_50_ values for HEK293F-expressed VHHs for neutralization of SARS-CoV-2, we selected the VHH-IgG1 Fc fusion, C5-Fc, as the transgene to insert into the configurable expression chassis for RNA-programmed CRISPR/Cas9 delivery into the germline of *S. mansoni*. Figure 3 diagrammatically depicts the transgene, its components and expression product, the CRISPR knock-in strategy, including orientation of the promoter, signal peptide, C5-Fc coding region and amino acid sequence, dimerization of the VHH endowed by the IgG1 Fc fusion partner, and terminator motif (Fig. 3a–d), along with potential knock out as well as knock in alleles following transfection of the schistosome egg with the donor transgene and RNPs (Fig. 3e, f). Supplementary Fig. 3 provides the donor transgene sequence, while Supplementary Table 1 lists the nucleotide sequences of the guide RNAs and oligonucleotide primers deployed in the study.

### Transgene construct encoding the C5-Fc anti-SARS-CoV-2 antibody

For insertion into the *S. mansoni* genome, we targeted a site in Genome Safe Harbor 1 (GSH1) on chromosome 3 of *S. mansoni*^12^ and deployed three overlapping guide RNAs for programmed CRISPR/Cas9 knock-in, a target site and tactics that we had previously shown to be both not apparently harmful to the targeted schistosome egg and effective for insertion of a reporter gene into *S. mansoni* genome^12^. To prepare a synthetic transgene construct encoding the C5-Fc anti-SARS-CoV-2 antibody, we included short (50 nt each) left and right side homology arms, and the promoter and terminator of an endogenous ubiquitin gene of *S. mansoni*, Gene ID, Smp_335990, UBB protein (WormBase Parasite, version WBPS19)^25–27^ to drive expression of the transgene (Fig. 3a, b). The rationale for using this promoter was that using an endogenous, ubiquitously expressed gene promoter (like ubiquitin) has advantages, especially in non-model organisms^28,29^, including broad, constitutive expression in tissues, cells and developmental stages^30^. Indeed, based on transcriptomics data for this schistosome, the Smp_335990 gene is strongly, ubiquitously, and stage-independently expressed^31,32^. We anticipated its compatibility with native transcription machinery since an endogenous promoter would be likely to be recognized and accurately regulated by the helminth’s own transcription machinery (transcription factors, chromatin context) and this could be expected to reduce the risk of failure or silencing. Non-endogenous – viral or foreign – promoters can trigger epigenetic silencing, especially over time or in specific tissues^33^. The promoter and terminator sequences of Smp_335990 represent tractable and not overly large regulatory elements for donor constructs^12,26^ and, indeed, we had previously used the ubiquitin promoter and its terminator in transgene constructs for knock-in experiments, with Cas9 and Cas12a, encoding a GFP reporter, inserted at the same GSH1 site^26^.

The secretory signal peptide from the hookworm protein, ASP2, known to actively drive secretion of diverse helminth proteins in mammalian cell lines^34^, served to direct the nanobody fusion protein into the schistosome’s secretory pathway. Translation initiation is a major rate limiting step in protein synthesis and given the positive impact of translation of the amino acids encoded by codons 3 to 5^35^, we preemptively inserted two extra residues, Lys-Leu, at amino acid positions 3 and 4 of the signal peptide, MV*KL*VLVPLLALLAVSVH, aiming to improve protein synthesis of the transgene-encoded nanobody and, in turn, secretion of the recombinant C5-Fc fusion protein (Fig. 3c, d).

Our experimental approach had two strategic components. First, using *S. mansoni* as a biological foundry exploited the male schistosome’s long-term survival to enable continuous in situ production of the therapeutic transgene product. Second, fusing the C5 VHH to the Fc region of human IgG1 induced dimerization, yielding a bivalent antibody with two identical antigen-binding sites to enhance SARS-CoV-2 neutralization (Fig. 3d). We leveraged Fc-induced bivalency to amplify the potency of the parental VHH through the avidity effect. – a key advantage for targeting viruses with repeating surface antigens like the SARS-CoV-2^36^. Indeed, recombinant C5-Fc expressed in HEK293F cells was >30-fold more potent than C5 alone in the same system (Fig. 2). Additionally, the Fc domain promotes neonatal Fc receptor (FcRn)-mediated recycling, extending serum half-life and improving tissue distribution^37^. Finally, the Fc domain confers effector functions, including antibody-dependent cellular cytotoxicity and phagocytosis that could aid in the detection and clearance of virus-infected cells^38,39^.

### Derivation of the C5-Fc line and propagation through intermediate snail and definitive mammalian hosts

**Fig. 4:**
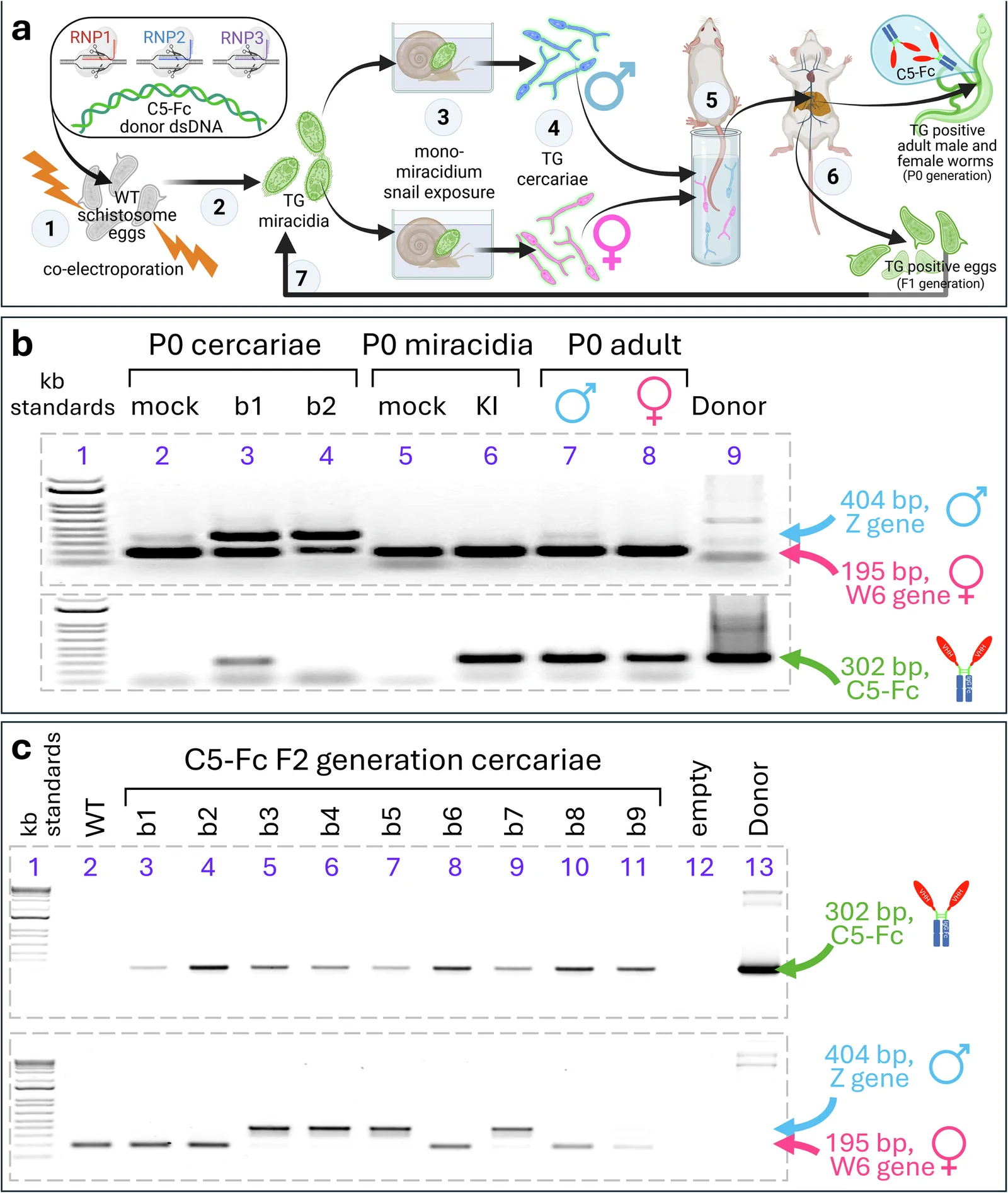
Derivation of the C5-Fc transgenic line. **a** Schematic overview of Schistosome transgenesis. *Schistosoma mansoni* eggs were transfected by electroporation with three RNPs targeting the genome safe harbor site GSH1 on chromosome 3 of *S. mansoni*^12^ and a double-stranded (ds) DNA donor encoding the C5-Fc fusion protein (1). Snails were exposed to miracidia that hatched from the eggs using mono-miracidial infection^40^ (2-3), mice infected with transgenic cercariae shed from the snails (4-5), and adult P0 schistosomes and F1 generation eggs recovered from the mice and investigated (6). F1 eggs hatched and used to perpetuate the F2 generation (7). **b** Representative PCR results – upper panel, PCR to determine sex; lower panel, PCR to determine presence or absence of the transgene – size standards (lane 1), cercariae from mock transfected controls (lane 2), cercariae from two discrete snails (b1, lane 3 and b2, lane 4 – both of which were identified by PCR as male) in the CRISPR KI group, eggs/miracidia of the P0 generation (mock treated control, lane 5; CRISPR KI group, lane 6), P0 adult male and female worms (lanes 7, 8), and the donor DNA plasmid (PCR positive control), lane 9. Note that of the cercariae shown in lanes 3 and 4, only lane 3 cercariae were transgene-positive; lane 4 cercariae were transgene negative. **c** Representative findings for the F2 generation of C5-Fc schistosomes. Lanes 3 to 11 each show PCR results for the presence of the transgene (upper gel) and the sex (lower gel) of cercariae sampled from cercariae shed from individual snails; all were transgene positive, five were female, four were male. Other lanes: lane 1, size standards, lane 12, no sample, lane 13, donor plasmid DNA (PCR positive control). With respect to panels 4b and 4c, PCR analysis for the presence of the C5-Fc transgene showed 3/48 positive snails in P0, 7/48 in F1, and 33/34 in F2. PCR assays performed on cercariae shed by individual snails were considered to be independent, biological replicates within each filial generation. Source data and uncropped gel images are provided as a Source Data file. Figure 4a and graphic inserts in 4b and 4c were created in BioRender. Smout, M. (2026) https://BioRender.com/xq7681u.

Figure 4a schematically displays the technical steps used to derive the C5-Fc line of transgenic *S. mansoni*. In vitro laid (immature) schistosome eggs (IVLEs) were transfected by electroporation with three overlapping RNPs targeting GSH1 on chromosome 3 of *S. mansoni*^12^ along with a 5′-modified-double stranded DNA donor encoding C5-Fc. A polymerase chain reaction (PCR)-based screen using transgene specific primers of a sample of the hatching population of these eggs and miracidia confirmed the presence of the C5-Fc transgene (302 bp amplicon; Figs. 4b, c and 4004 bp amplicon; Supplementary Fig. 4e). Forty-eight *Biomphalaria glabrata* snails were each exposed to a single miracidium hatched from this cohort (monomiracidial infection^40^). By 12 weeks later, C5-Fc-positive cercariae had been shed from three of the 48 snails (6.3%), whereas 16 snails shed wild type cercariae. Male cercariae shed from one of those three transgene positive snails, female cercariae from a second, and the third died before yielding enough cercariae for mouse infections (Supplementary Fig. 4b).

The sex of cercariae shed from each snail was determined using a competitive PCR^41^; a 404 bp amplicon specific for the *rpS4* gene, located only on the Z chromosome – confirming the sex of the cercariae as male, and a 195 bp amplicon of W6 repetitive element for the W chromosome, confirming the sex of the cercariae as female. In this PCR, a higher Z-specific gene: a W-localized repetitive element copy ratio results in the presence of both 404 and 195 bp amplicons and indicates male DNA, whereas the presence of only the 195 bp amplicon indicates female DNA. Figure 4b shows representative PCR results including the demonstration of both P0 transgenic and WT cercariae from the monomiracidial infection of the snails.

Each of six mice was exposed first to ~90 male cercariae and then a few weeks later (when the female cercariae became available) to ~100 female transgene (TG)-positive cercariae, aiming to establish a sexually reproducing population of P0 generation transgenic schistosomes within each mouse. About eight weeks after the infection with the female cercariae, the mice were euthanized, and adult schistosomes perfused from the portal circulation. Among the perfused worms, we observed up to five paired adult schistosomes among the ~20 worms obtained from each mouse. These adult schistosomes represented the foundation P0 generation of the C5-Fc line. Concurrently, we also recovered the schistosome eggs from the livers of the mice, the eggs representing the F1 generation. Samples of the adult P0 female and male worms were positive for the C5-Fc transgene (Fig. 4b) as were samples of the F1 generation eggs (Supplemental Fig. 4. b-e).

Eggs of this F1 cohort, isolated under aseptic conditions by collagenase digestion of all six mouse livers^42^, pooled into a single digest, were induced to hatch. Again, 48 snails were individually exposed to miracidia hatched from this transgene positive cohort of F1 generation eggs. In turn, shedding of cercariae was periodically induced from the snails from five weeks after exposure. By 12 weeks, C5-Fc-positive cercariae had been shed from seven of the 48 snails (14.6%); five of these snails shed female cercariae and two shed male cercariae. Over several weeks from the first appearance of transgenic F1 generation cercariae onwards, and as sufficient numbers of cercariae became available, six mice were each infected with ~100 female and ~100 male cercariae of the F1 generation of the nascent C5-Fc line. In turn, eggs were recovered from the livers of these mice, which also were C5-Fc positive, and thus represented the F2 generation.

The F2 generation was similarly propagated, with miracidia hatched from F2 eggs. PCR of F2 cercariae from individual snails infected by these F2 generation miracidia showed a 97% transgenic rate – 33 of 34 snails shed transgenic F2 cercariae (Fig. 4c, lower gel). Figure 4c (upper gel) shows PCR screening of a representative batch of cercariae from nine of these 33 snails; four snails produced male and five produced female cercariae (Fig. 4c, lower gel). Amplicons of Z and/or W chromosome- localized markers provided a DNA integrity control.

### C5-Fc secreted by TG schistosomes potently neutralizes SARS-CoV-2 in vitro

**Fig. 5:**
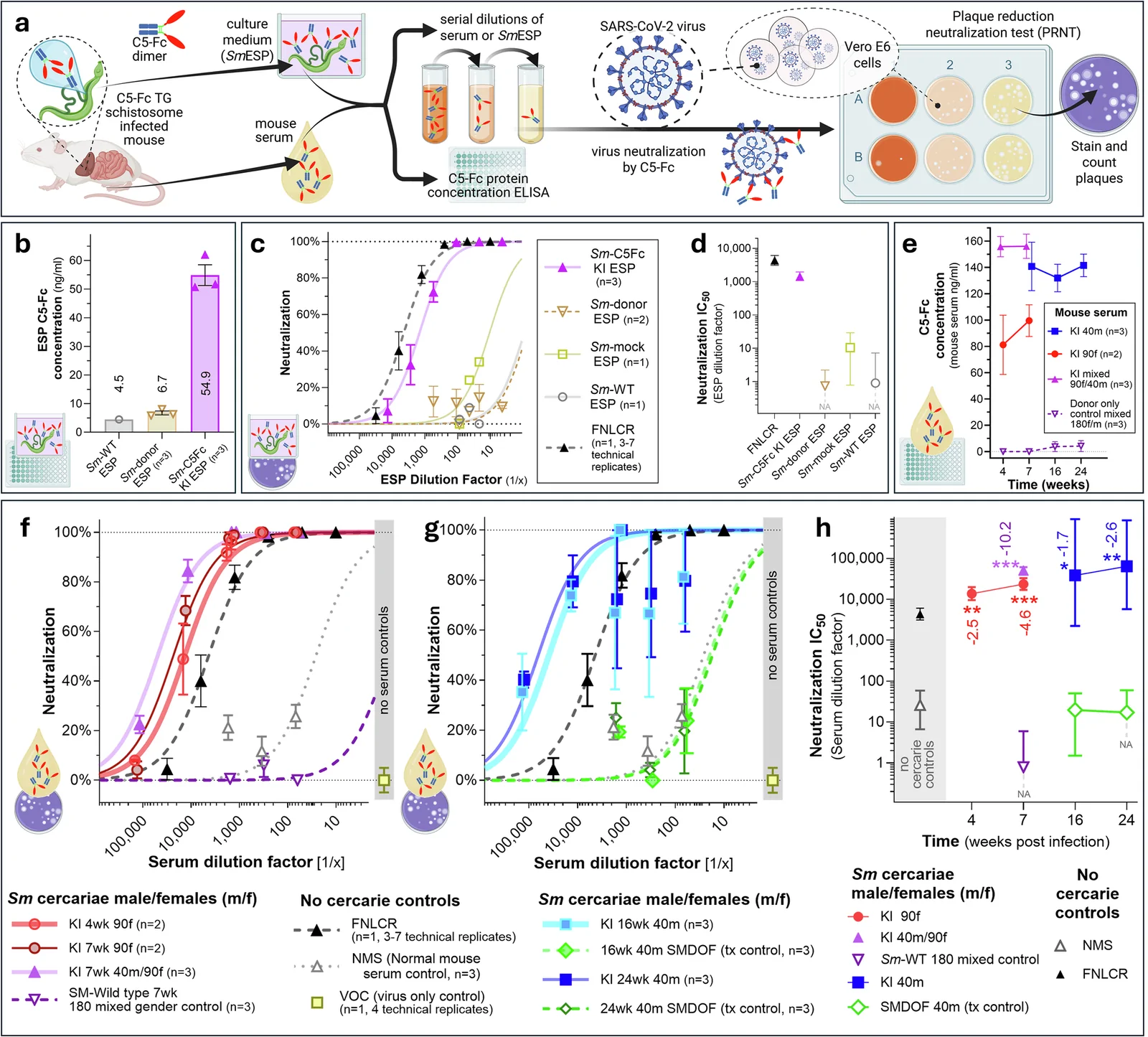
C5-Fc secreted into the circulation of mice infected with transgenic schistosomes potently neutralized SARS-CoV-2. **a** Schematic showing experimental process with assessment of neutralization of SARS-CoV-2 by infected mouse sera or transgenic schistosome ESP by plaque reduction neutralization test (PRNT). **b** ESP C5-Fc concentration. C5-Fc secreted by transgenic worms into ESP was quantified using a capture assay plotted as mean ± SEM. **c**, ESP PRNT neutralization (mean ± SEM) plotted against dilution factor of ESP. The nanobody in ESP achieved ~100% virus neutralization whereas ESP from control groups of schistosomes (donor only, mock treated, wild type [WT]) did not. FNLCR, convalescent patient serum standard. **d** ESP neutralization IC_50_ from panel c expressed as dilution factor. **e** C5-Fc concentration in mouse serum. C5-Fc secreted by transgenic worms into mouse blood, and serum collected 4–24 weeks post-infection. C5-Fc concentration was quantified using a capture assay plotted as time vs mean concentration ± SEM. **f** and 16-24 weeks (**g**) displayed dose-dependent in vitro neutralization of SARS-CoV-2^20^. PRNT neutralization (mean ± SEM), plotted against dilution factor of mouse serum. **h** Mouse serum neutralization IC_50_ from panels **f** and **g** expressed as serum dilution factor. Sera in panels **e**, **f**, **g** and **h** were from mice exposed to the P0 or the F1 generation of the C5-Fc transgenic line. Statistical comparison using extra sum-of-squares *F* test with Holm-Bonferroni multiple comparison correction comparing log(IC_50_) values against the FNLCR control: *P ≤ 0.05; **P ≤ 0.01; ***P ≤ 0.001. Exact log *P* values listed next to asterisks. **5b**,** c**,** e**,** f,** and** g:** individual mice used as biological replicates were specified as “*n* = X” values; technical replicates as listed. **5 d** and** 5 h**: IC₅₀ values calculated from previous panels using log (inhibitor) vs normalized response with Hill slope = -1; data points represent best-fit values for IC₅₀ with 95% confidence interval bars. Grey dashed bar labelled with “NA” represents an undefined 95% confidence interval. All panels**:** Source data including test statistics are provided as a Source Data file. As in Fig. 2, the SARS-CoV-2 USA-WA1/2020 isolate was used here. Panel 5a and additional graphic inserts for panels 5b-g created in BioRender. Smout, M. (2026) https://BioRender.com/e2eodov.

C5‑Fc produced by transgenic *S. mansoni* was detected in both excretory/secretory products (ESP) and sera of infected mice and mediated potent neutralization of the SARS‑CoV‑2 virus. Figure 5a provides a schematic overview of our assessment approach. Specifically, a quantitative capture ELISA revealed the presence of C5-Fc in ESP from pools of adult, mixed sex schistosomes, confirming secretion of C5‑Fc by the schistosomes at a concentration of 54.9 ± 4 ng/ml culture medium (Fig. 5b). The ELISA detected much lower C5‑Fc levels in the ESP of two negative-control worm groups: worms transfected with donor transgene but without RNPs, or mock‑transfected worms (Fig. 5b). Thereafter, we examined whether ESP containing the secreted C5-Fc could neutralize SARS-CoV-2 virus, as described for C5-Fc in the original report for recombinant C5-Fc^18^. We assessed neutralization of SARS-CoV-2 virus using the PRNT assay, in which the FNLCR was included as the positive control (Fig. 5c, d). ESP and FNLCR were diluted up to 31,250-fold, and neutralization levels plotted alongside the negative control ESP groups. ESP from the transgenic schistosomes diluted 1/108 completely ( ~ 100%) neutralized SARS-CoV-2, with an IC_50_ of 1,443 dilution factor (DF), a neutralization capacity only modestly lower than that of FNLCR (4,345 DF). By contrast, the ESPs from each of three negative control schistosome groups including the donor transgene only (no RNPs) exhibited significantly less virus neutralization power and markedly lower IC_50_ values (Fig. 5c, d). However, among the negative controls, ESP from the donor transgene only did exhibit some anti-viral neutralization (IC_50_ value of 11 DF), which may have resulted from low level expression of episomal (non CRISPR integrated) transgene DNA.

Next, we sought to determine whether, as with ESP, C5-Fc was released into the blood of mice infected with transgenic C5-Fc line schistosomes. As with ESP, a capture ELISA was used. Remarkably, C5-Fc was present in readily detected levels in sera of mice collected from 4 to 24 weeks (81.2–156.1 ng/ml) after infection (Fig. 5e), confirming that transgenic schistosomes secreted the C5-Fc fusion protein into the blood of infected mice. Sera from mice infected for 4-7 weeks (Fig. 5f) and 16–24 weeks (Fig. 5g) with TG flukes potently neutralized SARS-CoV-2 in a dose-dependent fashion and displayed even greater potency than the positive control FNLCR human convalescent serum. Normal mouse sera or sera from mice infected with WT or donor-only control flukes did not substantially neutralize the virus (IC_50_ for these negative controls ranged from 0.8–26.1 DF). IC_50_ values calculated from TG schistosome-infected serum as dilution factors showed potent neutralization at all time points tested from 4 weeks after infection of mice infected with TG male only (16 wk, 38,395; 24 wk, 64,074 DF), female only (4 wk, 13,665; 7 wk, 23,429 DF) and mixed sexes (7 wk, 49,574 DF) (Fig. 5h). Sera sampled beyond 4 weeks after infection, where available, displayed a trend towards increasing neutralization with increasing duration of infection. IC_50_ values were significantly higher in sera from all mice infected with TG flukes than that for the FNLCR standard (4,345 DF) (*P* ≤ 0.05).

### Stability and maintenance of C5-Fc transgenic line

Finally, in addition to detailed information collected during the establishment of the transgenic line (above), the C5-Fc line was subsequently maintained by routine laboratory passage: naïve snails exposed to 6–10 miracidia and mice to 100–200 cercariae. Periodic sampling of genomic DNAs and total RNAs of adult stages during generations F3 to F9 confirmed transmission of the transgene while, concurrently, hatching of eggs recovered from mouse livers, motility and infectivity of miracidia and cercariae, presence of paired adult worms in the portal circulation at mouse necropsy, and hepatosplenomegaly and hepatic granulomas of host mice, confirmed the viability and vitality of the transgenic schistosome line. Supplementary Fig. 5 and Supplementary Movies 1, 2 provide representative gels and images representative of these findings among these later C5-Fc generations, including the salient finding by western blot that F8 generation transgenic schistosomes secreted the C5-Fc antibody into the circulation of the host mice (Supplementary Fig. 5f).

## Discussion

This report describes the genetic engineering of *Schistosoma mansoni*, a flatworm parasite of humans, for the production and secretion of a llama nanobody fused with the Fc domain of human IgG1. To achieve this, a synthetic gene encoding the anti-SARS-CoV-2 nanobody termed C5 was fused with the Fc to express an engineered antibody fragment targeting the spike protein of SARS-CoV-2. The construct was inserted into a genome safe harbor site (GSH1) within the parasite genome^12^. The chimeric construct was introduced into the genome of *S. mansoni* using multiple, overlapping CRISPR guide RNAs. The expression of this chimeric antibody was driven by a schistosome ubiquitin promoter and terminator, to ensure robust and sustained production within the cells and tissues of the parasite, notably those tissues that produce ES products that are released by the flukes into the bloodstream of the infected human host. Ubiquitin is generally expressed in most cell types and developmental stages, given its essential cellular role in protein homeostasis and turnover^43^.

During the COVID-19 pandemic, Huo et al. 2021^18^ described several VHHs obtained from a llama experimentally immunized with the spike protein of SARS-CoV-2. One of these VHHs, termed C5, overlaps the ACE2 epitope, the receptor for viral entry of human cells. C5 neutralizes the Victoria strain, an early isolate of SARS-CoV-2 and the coeval, highly transmissible Alpha strain (B.1.1.7 first Africa). Pertinently, administration of a C5-trimer via the respiratory route showed potent therapeutic efficacy in a Syrian hamster model of COVID and separately, provided effective prophylaxis^18^. Given the noted performance of C5 and C5-Fc, we used these VHHs in our present study and in addition included for comparison several other VHHs reported during the pandemic^19^.

There was a two-fold rationale behind our experimental approach. First, by utilizing *S. mansoni* as a biological foundry, we capitalized on the long-term survival of the male schistosome^44,45^ thereby facilitating the continuous production of the therapeutic product in situ. Second, the fusion of the C5 VHH with the Fc region of human IgG1 aimed to enhance the neutralizing activity against SARS-CoV-2 by inducing dimerization that, in turn, produces a bivalent nanobody with two identical antigen binding sites. Addition of an Fc domain to C5 would also slow the dissociation rate, which is particularly important for viruses with repeating surface antigens such as the spike protein of SARS-CoV-2. Indeed, recombinant C5-Fc expressed in HEK293F cells displayed an IC_50_ that was more than 30-fold better than that of C5 expressed in the same cell line (Fig. 2). Inclusion of the Fc domain also ensures recycling of the nanobody fusion through the neonatal Fc receptor, thereby improving its pharmacokinetic profile by extending the serum half-life and optimized tissue distribution. Finally, the Fc region also endows effector functions including antibody-dependent cellular cytotoxicity and phagocytosis^38,39^.

To establish a tractable approach, we tested several variables, including target-site prediction in the schistosome genome, overlapping guide RNAs targeting a region predicted to be a chromosomal GSH for transgene insertion, transgene construct size ( < 5 kb), promoter choice, donor DNA modification to extend half-life, and the developmental stage of schistosome eggs^11,13–16,46^. In vitro assays confirmed expression of a functional C5-Fc antibody in schistosomes, including binding specificity to spike protein and SARS-CoV-2 neutralization. In vivo studies in mice, an established rodent model of human schistosomiasis^47^, were then used to evaluate therapeutic efficacy in a physiological context. Together, these results support S. mansoni as a platform for sustained production of an anti-SARS-CoV-2 biologic.

For the knock-in protocol, we selected a 3598-bp dsDNA donor PCR amplicon with 50-bp homology arms (HAL/HAR) and 5′ AmC6 PEG10 modifications introduced via PEG10 AmC6-modified PCR primers to improve donor stability, suppress non-HDR repair, and facilitate insertion of multi-kilobase cargo at a GSH. We used three overlapping high-fidelity Cas9 RNPs (chemically modified, ATTO 550-labeled sgRNAs) delivered by a single square-wave electroporation pulse to IVLE (0–48 h), targeting zygotic and early embryonic stages while reducing mosaicism. These choices were informed by prior mammalian HDR studies and our earlier work in schistosomes^12,48,49^. Although these conditions yielded a positive outcome, systematic testing of alternatives, such as long ssDNA donors, plasmid donors, longer homology arms, Cas12a RNPs, HDR-promoting small molecules, and optimized electroporation settings, could improve knock-in efficiency and germline transmission in *S. mansoni*^50–53^.

Parental (female and male) P0 transgenic *S. mansoni* were established and used to infect mice; eggs from these mice produced the F1 generation, and miracidia from F1 eggs infected snails to propagate the C5-Fc line into the F2 generation (Fig. 4). The line was maintained for multiple subsequent generations while retaining the full life cycle, infectivity, and virulence of the parental NMRI strain, demonstrating a robust, tractable platform for molecular and therapeutic studies. Importantly, western blotting showed F8 parasites continue to secrete the C5-Fc antibody into the host bloodstream (Supplementary Fig. 5f), validating the GSH1 site on chromosome 3 as a reliable locus for future transgenic lines and establishing an entirely novel biological drug-delivery platform.

Given these outcomes, we speculate that lines of transgenic worms based on this exemplar provide the blueprint to generate a bioengineered human schistosome platform to constitutively and/or conditionally produce and deliver peptide/protein-based drugs within the human host. These findings also provide a roadmap to genetically manipulate schistosome biology and the host-parasite relationship. Heritable transgenesis in other helminths might now be sought by generating CRISPR knock-out and knock-in lines with informative loss- or gain-of-function mutation, as described in the roundworm *Strongyloides* for genes controlling host finding and developmental arrest^54–56^. Achieving heritable transgenesis requires efficient delivery of CRISPR to the germ line, which is technically challenging in schistosomes. Hermaphroditic flatworms such as liver flukes and tapeworms may be more tractable because CRISPR delivery to the egg should enable germline modification. Yet schistosomes, despite being dioecious, are uniquely amenable among neodermatan platyhelminths because a transgene introduced at the egg stage can be screened after infecting the intermediate snail host with a single miracidium: cercariae derived from a single miracidium are clonal and sex-homogeneous. Infection of mice with transgenic cercariae of both sexes generated in this way is expected to yield transgenic F1 offspring and permit establishment of stable transgenic schistosome lineages^49^. Notwithstanding our success with schistosomes with these methods, transgenesis approaches from the planarian *Macrostomum lignano* also may be useful in parasitic flatworms^57,58^.

Reproducibility of targeted knock‑ins can be variable across systems^59,60^. Although we report the findings of one primary electroporation experiment, prior work from our group and others indicates consistent outcomes: IVLE electroporation with overlapping Cas9 RNPs and modified dsDNA donors typically yields overall editing (indel+HDR) of ~20–30% and reporter‑positive rates among edited eggs of ~60–80% when electroporation is optimized (e.g., 125 V); higher voltages reduce hatch rates^12^. The lifecycle of *S. mansoni* (egg → egg) is ~three months under favorable laboratory conditions, and it took 9 –12 months from construct design for the C5-Fc transgene to obtain the F2 generation in this study, a constraint on performing many independent replicates. Finally, retroviral transduction of IVLEs (versus liver eggs) produces substantially higher transgene copy numbers in downstream cercariae and reliable germline transmission, albeit with random integrations^16^.

One practical use – among many – would be a worm-based delivery system, where experimental schistosome infection has been suitably modified to be subclinical/minimally harmful in humans, e.g., male only schistosome infection^4^, to deliver therapeutic benefit^2,61–64^. Indeed, this is contemplatable given infection models for *S. mansoni* and other helminth species are being trialed in volunteers with the goal of establishing safety and efficacy of the model for downstream investigation of new therapies to control schistosomiasis in endemic regions^4,40,65^. Although male-only schistosome infections may induce symptoms of acute schistosomiasis when applied in high doses, repeated infections are extremely well tolerated, in line with the natural immunoregulatory nature of helminths^66^. Both male and female single-sex schistosomes have been used in controlled human infection studies^4,66^, however, because our long-term goal is to use transgenic schistosomes in vivo as a drug delivery platform, male rather than female single-sex infections are safer, since the main pathology of schistosomiasis is caused by eggs released by female parasites. In parallel, clinical use of schistosome antigens is considered as novel immunotherapy for autoimmune disorders^67^. Indeed, transgenic^66^ schistosomes could serve to express recombinant platyhelminth proteins, which often fail to be expressed in heterologous systems and, if expressed, are not post-translationally modified in a native manner^68,69^. Controlled human hookworm infection is under investigation for inflammatory bowel disease and other autoimmune and metabolic diseases^70^, as is development of defined biologics sourced from several nematodes including hookworms and filariae^71^. Pertinent also is a recent report that exposure to a lung-migrating helminth protects against SARS-CoV-2 infection through macrophage-dependent T cell activation^7^.

With this reproducible method for generating transgenic schistosome lines, research into fundamental questions – such as determinants of host range and species specificity and how schistosomes locate their predilection sites in the human host – can now proceed more rapidly, enabling deeper investigation of schistosome (patho)biology. These findings will also accelerate translational efforts, notably deployment of helminths as drug delivery systems.

## Methods

### Ethics

Mice (female, Swiss Webster, 8 wk old) infected with *S. mansoni* (NMRI strain; origin Puerto Rico^72^) were obtained from the Schistosomiasis Resource Center (Biomedical Research Institute, Rockville, MD) within seven days of infection by cercariae (180 cercariae/mouse/percutaneous infection). The mice were housed in the vivarium facility of the Office of Animal Research, Washington University, which is accredited by the American Association for Accreditation of Laboratory Animal Care (AAALAC no. 000347) and has the Animal Welfare Assurance on file with the National Institutes of Health, Office of Laboratory Animal Welfare, OLAW assurance A3205, using a 12-hour dark/ 12- hour light cycle, 23 °C ambient temperature, and 50% humidity. All procedures employed were consistent with the Guide for the Care and Use of Laboratory Animals. The Institutional Animal Care and Use Committee of the George Washington University approved the protocol used for maintenance of mice and recovery of schistosomes, protocol numbers A2020-037 and A2034-074, as did the Animal Care and Use Review Office of the US Army Medical Research and Development Command, ACURO protocol number NIWC-8735.e003. (With regard to sex as a biological variable, we used only female mice as the schistosome host, reasoning that the establishment of the transgenic schistosome line in one host sex provided more control because it removed sources of biological variability including sex-based differences in immune response, hormone levels, parasite burden and pathology.)

### Transgene structure

The transgene is 3598 bp in length, comprised on the following components: Homology arms of 50 bp each at the left (homology arms left, HAL) and right (HAR) termini, matching the native GSH1 at the programmed CRISPR knock-in site; gene promoter and terminator, respectively, elements from the *S. mansoni* ubiquitin gene, flanking the left-and right-hand sides of the protein-coding sequence were included to drive transgene expression; and a signal peptide from a secreted protein of dog hookworm *Ancylostoma caninum* to drive secretion of a SARS-CoV-2 neutralizing antibody (termed C5)-IgG1 Fc fusion protein, bearing a hexahistidine affinity tag at the C-terminus (GenBank accession PX933511).

We have described the promoter and terminator sequences of the *S. mansoni* ubiquitin gene Smp_335990, previously^26^. This promoter is a compact, functionally dense regulatory element that likely underpins robust, constitutive expression. The terminator displays a composite of eukaryotic termination signals consistent with efficient transcriptional termination and co-transcriptional RNA processing. It includes a conserved polyadenylation hexamer (AAUAAA) and adjacent sequence context that would provide the canonical signal for cleavage and polyadenylation for mRNA stability and nuclear export. Together, the promoter and terminator architectures form a self-contained regulatory unit that is expected to promote reliable transcription initiation and faithful termination – attributes that underpin its selection here and explain the utility of *the S. mansoni* ubiquitin regulatory sequences for driving consistent, sustained transgene expression in the genome safe harbor site.

### Fusion protein (VHH-IgG1Fc) encoding gene

We included the signal peptide MVKLVLVPLLALLAVSVH of an abundantly secreted, SCP (sperm coating protein)-like protein, Genbank: RCN53414.1, of hookworms^73,74^ to direct the nascent C5–Fc fusion protein to the secretory pathway. We inserted two extra residues (Lys-Leu) at amino acid positions 3 and 4 of the signal peptide to improve protein synthesis of the transgene-encoded llama nanobody^35^. The secreted C5–Fc fusion comprises a SARS-CoV-2–binding VHH, named C5 fused via a serine-rich in-frame spacer to the human IgG1 Fc fragment. The C-terminal hexa-histidine tag was included to facilitate affinity purification. The predicted molecular mass of the secreted fusion protein of 377 amino acid residues is 37.1 kDa. Figure 3c shows the amino-acid sequence. The rationale for deploying this specific VHH-IgG1-Fc fusion protein was as follows. C5 VHH overlaps the ACE2 epitope and hence prevents the receptor binding protein of the virus from engaging the host cell receptor^8^. C5 neutralized both the Victoria strain and the contemporaneous, highly transmissible Alpha strain (B.1.1.7 first Africa) of SARS-CoV-2. Delivering a trimer of the C5 nanobody through the respiratory tract yielded robust therapeutic benefits in the Syrian hamster COVID-19 model and, independently, effective prophylaxis. In addition, C5 was similarly potent in the hamster following intraperitoneal administration, and fusing an anti-Middle East respiratory syndrome coronavirus (MERS-CoV) VHH nanobody to an IgG1 Fc fragment extended the half-life in vivo of that nanobody and ameliorated MERS-CoV disease in a challenge model^75^.

### Doubled stranded DNA donor template

The double stranded DNA was chemically synthesized and ligated into pUC, to yield pUC_Ubi-C5VHH-Fc-Ubi (Azenta Life Sciences). This construct included short homology arms of 50 bp in length^26^ corresponding to GSH1 at 571–622 nt (5´-homology arm) and 640–690 nt (3´-homology arm), respectively, flanking the in-frame expression cassette of the *S. mansoni* ubiquitin gene promoter (2,056 bp)^12^, an open reading frame coding for the signal peptide (51 bp), the C5 VHH, fused via a linker to human IgG1 Fc, and C-terminal His_6_ tag (i.e., 1131 nucleotides encoding 377 amino acid residues) and a truncated ubiquitin gene terminator (304 bp)^12,26^. Plasmid pUC_Ubi-C5VHH-Fc-Ubi served as template for PCR amplification using Platinum *Taq* DNA Polymerase (Thermo Fisher). Primers specific to the ubiquitin promoter and terminator including 50 nt of HA, with PEG10-AmC6-modified 5´ends^76,77^ (Supplementary Table S1) were prepared as follows. Ten µM of 5’-amine C6 primers (Integrated DNA Technologies, Inc. (IDT)) were incubated with 1 mM Bis-PEG10-NHS ester (BroadPharm) in borate buffer (Thermo Fisher) at 23 °C for 18 h. Primers were desalted and dissolved in Tris-buffer using a column (Bio-Spin 30, Bio-Rad, Hercules, CA). The 5’ modifications including an amine group, a C6 linker (AmC6) and polyethylene glycol (PEG10) treated-primers specific for the 5´ and 3´ termini of the homology arms to enhance the efficiency of the subsequent programmed transgene knock-in, by improving the stability of the donor DNA and suppressing non-HDR repair pathways^77^. The PEG10-5´AmC6-modified primers (Supplementary Table 1) in the presence of the polymerase amplified the donor template during thermal cycling of 94 °C, 120 s; 25 cycles of 94 °C, 30 s, 55 °C 30 s; and 72 °C, 5 min. Amplicons of the expected size, 3,598 bp, were visualized following electrophoresis through SYBR Safe (Invitrogen) stained agarose.

### CRISPR transgene knock-in to a genome safe harbor

Mice were euthanized seven weeks after infection, after which schistosomes were recovered by portal vein perfusion with 150 mM NaCl, 15 mM sodium citrate, pH 7.0. Approximately two hundred mixed-sex NMRI strain mature worms were cultured overnight in Dulbecco’s Modified Eagle Medium (DMEM) (Gibco) containing 10% heat-inactivated bovine serum (FBS) and 2X penicillin, streptomycin sulfate, amphotericin B solution (Corning Life Sciences). In vitro laid eggs (IVLE) were collected from 0 to 48 h after removal of the adult schistosomes from the mice^16^ and subjected to CRISPR transfection. A transgenic line of schistosomes was raised from these progenitors. CRISPR/Cas9 catalysed knock-in (KI) of a novel transgene encoding an anti-SARS-CoV-2 antibody (below) into GSH1 of the schistosome germline of IVLE was undertaken using three ribonuclear complexes (RNP) of Alt‑R HiFi *Streptococcus pyogenes* Cas9 nuclease (with nuclear localization sequences) and synthetic guide RNAs (Alt‑R CRISPR‑Cas9 sgRNAs) chemically modified for enhanced stability and ATTO‑550 fluorescently labeled to track electroporation (IDT). Three RNPs were used, one with each three overlapping guide RNAs (gRNAs) (Supplementary Table 1). The three gRNAs were located on the forward strand of GSH1 at nucleotide positions 605–624, 617–636, and 623–642, respectively, targeting nucleotide positions between 605 to 642 of the GSH1. We had previously established, by computational genome annotation and chromatin structure analysis, that this site, at location chromosome 3:13380432-13381848 (1,416 bp), represented an intergenic genome safe harbor site ^65^, which we termed GSH1^12^ and noted there that all three gRNAs lacked both off-target sites and self-complementarity.

Each RNP was complexed in the separate tube, with Cas9 and a single guide RNA at 1:1 ratio in 25 µl Opti-MEM medium; 10 μl of 1 μg/μl sgRNA (Opti-MEM as diluent) was mixed with 10 μl of 1 μg/μl Cas9 (in Opti-MEM) by gentle pipetting and incubated for 10 min at ambient temperature to allow the RNP to assemble. We had used this approach to successfully insert a gene coding for green fluorescent protein into GSH1 of *S. mansoni*^12,78^. IVLEs (above) were washed three times with 1×PBS before transfer into a electroporation cuvette (4 mm electrode gap, BTX, Holliston, MA) with Opti-MEM as the electroporation buffer. Each 25 μl of RNP along with the double strand DNA donor was immediately dispensed into the cuvette with the schistosome eggs, to a volume in the cuvette of 300 μl with Opti-MEM. Transfection of IVLE with CRISPR materials was undertaken using square wave electroporation (Electro SquarePorator ECM 830, BTX, Harvard Bioscience, Holliston, MA), with a single pulse of 125 volts for 20 ms^48,79^.

### Establishment of transgenic line of *S. mansoni*

Following electroporation, transfected eggs were transferred to culture medium supplemented with 20% FBS, as above. The transfected population of IVLE was cultured in DMEM supplemented with 20% FBS and 2x PSF for nine days until the miracidium had fully developed within the eggshell. At this point, the cultured eggs were exposed to water to induce egg hatching. Miracidia that hatched from the eggs represented the P0 generation of the transgenic line of *S. mansoni*, termed the C5-Fc line. Immature *B. glabrata* snails (shell diameter ~5 mm) were exposed to a single P0 generation miracidium, and snails maintained in aquaria containing aerated, artificial pond water^80^. The non-hatched eggs and remaining miracidia were pooled and retained for downstream analysis, aiming to confirm targeted integration in the genome of the C5-Fc transgene. Integration of the C5-Fc transgene targeted to GSH1 of the *S. mansoni* genome of pools of cercariae shed from individual snails was confirmed by PCR. Sex of the cercariae was confirmed by PCR using the same pool of cercarial genomic DNA, after which 8-wk old female Swiss Webster (CFW) outbred (albino, strain code 024) mice (Charles River Laboratories) were exposed to additional cercariae shed from the same snail(s). For the parental (P0) generation of the nascent line of transgenic worms, each mouse was exposed by the tail method^81^ to ~100 female and ~90 male cercariae, ≤200 cercariae in total per mouse. Eggs representing the F1 generation of the C5-Fc line of transgenic schistosomes were recovered from livers of the mice^42^ ~ 80 days post-exposure of the mice to the P0 cercariae. The presence of the C5-Fc transgene in miracidia hatched from this cohort of eggs was confirmed as above, before generating the F2, in like fashion as for the F1 generation. Blood was sampled from the tail veins of mice at intervals, after which the mice were euthanized, and mouse livers were removed, schistosome eggs recovered from the livers, and adult worms retrieved from the portal circulation^42^. In addition, infection of additional mice with either female or male sex C5-Fc worms was undertaken for up to 24 weeks to monitor levels of the anti-SARS-CoV-2 antibody in the peripheral circulation over time. Sera sampled during the interval from 4 to 24 weeks after infection were evaluated for the presence of the C5-Fc VHH and in a plaque reduction neutralization test against SARS-CoV-2. Individual mice represented biological replicates. Serum was sampled from each mouse infected with female C5-Fc schistosomes (*n* = 2), mice infected with male schistosomes (*n* = 3), and those infected with both female and male schistosomes (*n* = 3). Controls comprised mice infected with wild-type S. mansoni (*n* = 3) and mice infected with transfection-control schistosomes (donor transgene only; *n* = 3).

F3 and later filial generations until F9 of the line of C5-Fc schistosomes were maintained using routine approaches to laboratory maintenance of *S. mansoni*, including exposing naïve snails and mice each to 6–10 miracidia and 100–200 cercariae, with a mixture of both sexes of the schistosome, respectively. Blood samples were collected at necropsy about eight weeks after infection, mouse livers were removed, schistosome eggs recovered from the livers, and adult worms retrieved, as above. As for the P0, F1 and F2 schistosomes, the vertical transmission and presence of the C5-Fc transgene in these later generations was monitored and confirmed by PCR was confirmed PCR presence. In addition, the presence of the C5-Fc VHH in the blood of the mice was assayed by western blotting including blood from mice infected with the F8 line of transgenic schistosomes.

### Nucleic acids

Genomic DNAs from IVLEs, egg-harvested from *S. mansoni-*infected mouse liver (LE = “liver eggs”) or mature worms were extracted as described^12^. Briefly, schistosomes (eggs, miracidia, cercariae, adult worms) in ~100 to 300 μl DNA/RNA Shield solution (Zymo Research) were triturated using a motor-driven homogenizer fitted with a disposable pestle and collection tube (Biometer II, Bunkyo-ku). DNA was isolated the homogenate; 250 μl DNAzol ES (Molecular Research Center, Inc.) was dispensed into the homogenate, and DNA recovered according to the manufacturer’s protocol. Total RNA was isolated from a similar homogenate; 250 μl RNAzol ES (Molecular Research Center, Inc.) was dispensed into the homogenate, and RNA recovered according to the manufacturer’s protocol. Yields and purity were assessed and quantified by spectrophotometry (NanoDrop One Spectrophotometer, Thermo Fisher Scientific), based on the ratios of absorbance at 260/280 and 260/230 nm^82^.

### Screening for integration of C5-Fc transgene in schistosome genome

Integration of the C5-Fc donor transgene in developmental stages of schistosomes was monitored by PCR analysis of genomic DNAs from schistosomes. The PCR amplification catalyzed by GoTaq G2 DNA polymerase (Promega) used a pair of primers; forward primer C5VHH-F, located on the anti-SARS-CoV-2 C5 VHH coding sequence and reverse primer FcIgG-1-R, targeting the human IgG1 Fc coding sequence (Fig. 3f; Supplementary Table 1), with cycling conditions of 95 °C, 2 min, 30 cycles 94 °C, 15 sec, 58 °C 30 s, 72 °C, 60 s. Amplification products were separated through 1% agarose by electrophoresis and stained with SYBR Safe, with predicted amplicon of 302 bp (Figs. 3f, 4b, 4c). To examine the mRNA expression of C5-Fc, total RNAs extracted from adult schistosomes were exposed to DNase to eliminate residual genomic DNAs and transcribed into cDNA using the Maxima First Strand cDNA synthesis kit with DNase (Thermo Fisher Scientific). The qPCR was performed using the GoTaq G2 DNA polymerase (cat no. M7841, Promega, Madison, WI) with the transgene specific primers (above) with expected amplicon at 302 bp. *S. mansoni* GAPDH (Smp_056970) served as the reference gene, using *Smgapdh*-specific primers (Supplementary Table S1) for an expected amplicon of 285 bp in length. PCR cycling conditions were 95 °C, 120 s, 35 cycles 94 °C, 30 s, 60 °C, 30 s, 72 °C, 60 s, after which amplification products were examined as above.

Integration of the C5-Fc transgene at the programmed target site at GSH1 in the F1 transgenic population of schistosomes was confirmed by direct PCR analysis of genomic DNAs from the F1 generation adult worms recovered from mice infected with F1 generation cercariae. Primers specific for upstream, 5’-Out-F and downstream, 3’-Out-R of the programmed target site in GSH1 (Fig. 2f; Supplementary Table 1). Thermal cycling with Platinum Taq DNA polymerase (Thermo Fisher) consisted of 94 °C initial denaturation, then 40 cycles of 94 °C for 30 s, 60 °C for 30 s and 72 °C for 4 min to amplify the integrated transgene at the GSH1 site (predicted amplicon: 4,004 bp). Sexing of schistosomes was accomplished using a sex determination multiplex end point PCR, reported by Chevalier et al.^41^, which targets the female-specific W6 repetitive element (GenBank HQ880215) and the Z chromosome-linked *rpS4* (40S small ribosomal subunit protein S4) gene (Smp_011570.1), using W6-specific primers (amplicon, 195 bp) and Z chromosome-specific *rpS4* gene primers (amplicon, 404 bp) (Supplementary Table 1). Except for the sexing primers^41^, all other primers were designed using SnapGene version 6.0 or later (GSL Biotech LLC, San Diego, CA).

### Excretory/secretory products (ESP) of schistosomes

Isolation of ESP from adult schistosomes of mixed sexes following exposure to CRISPR and donor transgene was undertaken as described^83^. ESP collected from three separate wells of six-well plates containing cultured adult schistosomes represented discrete biological replicates. Protein concentration in ESP was ascertained using the using the bicinchoninic acid method (Pierce BCA Protein Assay Kit, Thermo Fisher) and concentrated ESP aliquoted and stored at -80 °C.

### ELISA for detection of C5-Fc nanobody secreted by transgenic worms

Our custom ELISA used SARS-CoV-2 nucleocapsid/spike protein (RBD) (Invitrogen RP-87706) and a rabbit anti-camelid (camel and llama) VHH-conjugated to horse radish peroxidase [HRP] cocktail (GenScript A02016) to capture *S. mansoni* secreted C5-Fc VHH in ESP and mouse sera. Plates were coated with 1 μg/ml recombinant SARS-Cov-2 nucleocapsid/spike protein at 50 μl/well, blocked, and probed with two-fold serial dilutions of positive control, 1000 ng/ml to 0.48 ng/ml recombinant C5-Fc produced in HEK293F cells, or probed with ESP or with mouse serum diluted 1:10, followed by the rabbit-anti-VHH cocktail-HRP conjugate at 1: 4000 dilution (MonoRab, GenScript catalog A02016) and developed with 3,3’,5,5’-tetramethylbenzidine ELISA substrate (Abcam Inc, Waltham, MA). Assays were performed in duplicate or triplicate with optical density at 450 nm measured using a spectrophotometer (Omega Polar Star, BMG Labtech, Cary, NC). Concentrations were determined by interpolation from a cubic polynomial least squares fit C5-Fc standard curve plotted in GraphPad Prism 10.6.

### Western blot

Proteins including mouse sera recombinant C5-Fc VHH and pre-stained protein standards (SeaBlue Plus2, Thermo Fisher Scientific) were resolved by SDS–PAGE through 4–12 % polyacrylamide gel (Bolt Bis-Tris WedgeWell, Invitrogen,) and transferred to polyvinylidene difluoride membrane (Bio-Rad) using a semi-dry transfer system at 1.3 A, 25 V for 7 min (Trans-Blot Turbo System, Bio-Rad). The membrane was blocked for overnight at 4 °C Tris-buffered saline pH 8.0 with 0.1% Tween-20 (TBST) containing 5% nonfat dry milk, then incubated overnight at 23 °C with an HRP-conjugated rabbit polyclonal IgG antibody detecting 6X His tag (Abcam, catalog no. ab1187), diluted 1: 5000 in TBST with 1% nonfat dry milk. Following three 5 min washes in TBST, blots were developed using an enhanced chemiluminescence substrate (SuperSignal West Pico Plus, ThermoFisher) and imaged on a signal detector, with exposure time adjusted to avoid signal saturation (ChemiDoc Imaging System, Bio-Rad).

### Microscopy

Zeiss Primovert (Carl Zeiss LLC, Thornwood, NY) was used for routine brightfield observation of schistosome developmental stages. Fluorescence imaging was performed on a Zeiss Axio Observer A1 inverted microscope fitted with an AxioCam 208 color camera (Carl Zeiss). Schistosome eggs labeled with ATTO 550–RNPs were imaged with a Zeiss 20× LP Plan Neofluar 20×/0.4 Korr Ph1 Ph2-M27 objective. Excitation used the microscope’s 540–560 nm LED/filter set and emission was collected through a 570–590 nm band-pass filter. Images were acquired at 12-bit depth, 1× binning, pixel size 6.45 µm, exposure 200–500 ms (adjusted to avoid saturation) and saved as TIFFs for downstream analysis. Short videos and still images were captured using the rear-facing camera of an iPhone 16, handheld. Up to 9 × magnified images were obtained by using the camera’s optical/combined digital zoom, focused manually, and captured in high-resolution photo mode; short videos ( ≤ 30 s) were recorded at 1080p/30fps to document motion. Images and videos were transferred to a computer and processed uniformly: stills were cropped and adjusted for exposure/white balance using [image software], and videos trimmed without altering frame rate.

### Mammalian cell expression of recombinant VHHs

The following VHHs targeting the spike protein of SARS-CoV-2 were selected from the public domain for expression because they have been shown to neutralize the virus: VHH-C5^18^ and VHH-E and VHH-V^19^. VHH-C5 displayed the most potent neutralizing capacity with pseudotyped virus (Supplementary Fig. 2) and SARS-CoV-2 (Fig. 2), so we constructed a C5-Fc fusion protein with the Fc domain of human IgG1 (Fig. 3). We included a modified version of the mouse IgG heavy chain signal peptide, MVKIWVFLFFLSVTTGVHS, where the first four residues were modified to improve secretion, as described elsewhere^35^. The recombinant proteins included a C-terminal hexa-histidine tag. Nucleotide sequences encoding VHH-C5-Fc, VHH-C5, VHH-E and VHH-V, which includes the mouse IgG heavy chain signal peptide, have been assigned GenBank accessions PX933512, PX933513, PX933514, and PX933515, respectively. Recombinant VHHs were produced in Expi293F HEK (human embryonic kidney) 293 cells (catalog no. A14527, Thermo Fisher Scientific) via transient transfection using the ExpiFectamine^TM^293 transfection kit (ThermoFisher Scientific). Briefly, a suspension of HEK293F cells were grown in culture (30 ml volume) to a density of 2 × 10^6^ cells/ml and then transformed using 30 μg of purified pcDNA3.1_C5-Fc plasmid DNA by lipofection. Transformed cells were maintained at 37 °C in 5% CO_2_ on a shaking platform for 72 h. The culture supernatant was harvested by centrifugation, sterile filtered using 0.22 μm Millipore syringe filters, after which the 6xHis tagged VHH was recovered by immobilized metal-ion affinity chromatography using a His-Trap excel nickel column (Cytiva). Following elution into 500 mM imidazole, fractions were assessed by Coomassie blue-stained SDS-PAGE and those fractions containing purified VHH (as assessed by molecular mass) were pooled, concentrated and buffer exchanged into Dulbecco’s PBS using a 3 kDa Amicon Ultra centrifugal concentrator (Merck). Purified VHHs were analyzed for endotoxin content using the *Limulus* Amebocyte Lysate assay (Thermo Fisher Scientific) and concentration determined using the BCA assay (Thermo Fisher Scientific).

### Lentiviral pseudovirus assay

Neutralization performance of the recombinant VHHs was assessed using a lentiviral pseudovirus neutralization assay^84^. Pseudotyped lentivirus (LV) that encoded the SARS-CoV-2 spike glycoprotein receptor binding domain (Wuhan, GenBank MN908947) was produced via lipofectamine LTX (Thermo Fisher) transfection of HEK293T cells (Millipore Sigma, St. Louis, MO, catalog no. 12022001-1VL). The SARS-CoV-2 pseudotyped virus was based on the HIV-1 lentiviral packaging system incorporating a luciferase reporter. Luciferase activity of virus stock was determined in HEK293T cells stably expressing human ACE2 (ACE2-HEK293T) by measuring relative luciferase units (RLU). VHHs or heat-inactivated NISBC (20/130) were serially diluted 5-fold in DMEM and mixed with 50,000 RLU of virus and then incubated at 37 °C for 60 min. The serum–virus mixture was added to the seeded ACE2-HEK293T cells (1 × 10^4^) in a 96-well plate in a total volume of 150 μl, after which the cells were maintained at 37 °C, 5% CO_2_ for 72 h. At that point, culture medium was removed, replaced with 100 μl luciferase substrate (BioGlo, Promega, Cambridge, UK) and left for 5 min to lyse the cells and allow the luciferase reaction to proceed. Luminescence was measured in duplicate samples in a microplate reader (Varioskan, Thermo Fisher Scientific) with neutralization determined as the percentage RLU of the negative (immune serum-free) virus control.

### Plaque reduction neutralization test

PRNT was performed to evaluate the neutralization efficacy of the samples in vitro^21,85^. In brief, 6-well plates of Vero E6 cells (ATCC catalog no. CRL-1586) were seeded a day earlier to achieve next day confluency. Serial dilutions of samples were prepared using DMEM supplemented with 2% fetal bovine serum (FBS) (VWR, 97068-085) as a diluent in a 96-well, deep-well plate (Fisher Scientific). Virus (SARS-CoV-2 USA-WA1/2020 isolate from BEI, https://www.beiresources.org/Catalog/animalviruses/NR-52281.aspx)^23^ was added to each well for a 1:1 ratio of virus to sample volume, targeting 100 plaque forming units (PFU) for the 6-well plate, i.e., 325 μl of virus at a titer of 1 × 10^3^ PFU/ml added to 325 μl of sample; 200 μl of each mixture was used per well for plaque assay. Plates were incubated at 37 °C, 5% CO_2_ for 60 min. Thereafter, medium was aspirated from the Vero E6 cells and 200 μl of each virus-sample mix was added to the appropriate wells in triplicate as technical replicates for each sample and concentration. Biological replicates included discrete batches of schistosome ESPs and sera from individual mice. A limited volume of serum from one of the knock-in male worm-infected mice allowed us to include only two biological replicates for the 1/1,300, 1/13,000, and 1/130,000 dilutions.

The plates were incubated at 37 °C, 5% CO_2_ for 60 min with gentle agitation at 15 min intervals. A 1:1 mixture of 2.5% Avicel RC-591 (DuPont Nutrition, Newark, DE) and 2X Modified Eagle Medium (MEM) (Gibco, 11-935-046) was added to each well as a semi-solid overlay^86^. The plates were incubated for a further 48 h after which the overlay was removed, the wells fixed and stained with crystal violet/ formalin. The plates were rinsed, plaque numbers counted, and viral titer calculated and compared to the diluted virus only control to determine the neutralization efficacy of the treatment.

GraphPad Prism 10.6 was used to visualize the neutralization dose response curves and calculate IC_50_ values with log(sample dilution factor) vs normalized percent neutralization values. Comparisons were undertaken with extra sum-of-squares *F* test with Holm-Bonferroni correction comparing log(IC_50_) values against the FNLCR control: *P ≤ 0.05; **P ≤ 0.01; ***P ≤ 0.001.

### Reporting summary

Further information on research design is available in the Nature Portfolio Reporting Summary linked to this article.

## Supplementary information

Supplementary information

Description of Additional Supplementary Files

Supplementary Movie 1. Hatching of Schistosoma mansoni eggs of F9 generation of the C5-Fc line, with the released miracidia actively in a beaker of pond water

Supplementary Movie 2. Biomphalaria glabrata snail during exposure to infection with miracidia of the F9 generation of transgenic S. mansoni.

Reporting Summary

Transparent Peer Review file

## Source data

Source data

## Data Availability

The nucleotide sequence data generated in this study have been deposited in the GenBank, specifically the nucleotide sequence of the donor transgene has been assigned GenBank accession PX933511 and the nucleotide sequences of four VHHs expressed in mammalian cells are assigned PX933512–PX933515. PX933511, https://www.ncbi.nlm.nih.gov/nuccore/PX933511, PX933512, https://www.ncbi.nlm.nih.gov/nuccore/PX933512.1/, PX933513, https://www.ncbi.nlm.nih.gov/nuccore/PX933513, PX933514, https://www.ncbi.nlm.nih.gov/nuccore/PX933514, PX933515, https://www.ncbi.nlm.nih.gov/nuccore/PX933515. Other accessions cited: AF089728.1, https://www.ncbi.nlm.nih.gov/nuccore/AF089728.1/RCN53414.1, https://www.ncbi.nlm.nih.gov/protein/RCN53414.1, HQ880215, https://www.ncbi.nlm.nih.gov/nuccore/HQ880215, Smp_011570.1, https://www.ncbi.nlm.nih.gov/nuccore/?term=Smp_011570.1, MN908947, https://www.ncbi.nlm.nih.gov/nuccore/MN908947. All other data, including source data for GraphPad Prism–based statistical analyses, curve fitting, and graphical representations, are available in the main text and/or the Supporting Information and/or the Source Data file.  Source data are provided with this paper.
