# Supplementary material for: Heritable transgenic schistosomes as a living platform for SARS-CoV-2 neutralizing antibody secretion
Source: Nat Commun. 2026 Aug 26;17:9052. doi: 10.1038/s41467-026-76465-9 (PMC13518888; doi:10.1038/s41467-026-76465-9)
Supplement: Supplementary file 1 [file 41467_2026_76465_MOESM1_ESM.pdf]

Supporting Information

Supplementary Figure 1, panels a – d, Gene and protein sequences of anti-SARS-CoV-2 VHH nanobodies. The nucleotide sequences and their encoded protein sequences of four VHHs produced in recombinant form in HEK-293 cells Nucleotide sequence followed by the protein sequence, with the latter annotated as follows: VHH (red colored font), hinge region (green), human IgG1 heavy chain constant region (blue), and 6x His tag (black).

a VHH-C5-Fc

GenBank accession PX933512

Synthetic construct SARS-CoV-2 spike protein binding camelid VHH-C5 nanobody with human IgG1 Fc region gene, complete cds

https://www.ncbi.nlm.nih.gov/nuccore/PX933512.1/

```
ATGGTCAAAATCTGGGTCTTTCTGTTCTTTCTGTCTGTCACAACTGGGGTCCATAGTCAGGTGCAGCTGGTCGAGTC
AGGAGGAGGTTCCGTGCAGGCTGGAGGCTCTCTGACCCTGAGCTGCGTGGCTTCCGGCGTGACACTGGGCAGACACG
CTATCGGATGGTTCAGGCAGGCTCCTGGCAAGGAGAGGGAGAGGGTGAGCTGTATCCGGACCTTCGACGGCATCACA
TCTTACGTGGAGAGCACCAAGGGCAGGTTTACAATCAGCTCCAACAATGCCATGAACACCGTGTACCTGCAGATGAA
CAGCCTGAAGCCCGAGGACACCGCCGTGTATTTCTGCGCTCTGGGCGTGACAGCCGCTTGTTCTGATAACCCCTATT
TTTGGGGCCAGGGCACCCAGGTGACAGTGTCTAGCGAGCCTAAGTCCTCTGACAAGACCCACACATCCCCACCTTCT
CCAGCTCCAGAGCTGCTGGGAGGCAGCTCCGTGTTCCTGTTTCCACCCAAGCCAAAGGATACCCTGATGATCAGCAG
AACCCCCGAGGTGACATGCGTGGTGGTGGACGTGTCCCACGAGGACCCCGAGGTGAAGTTTAACTGGTACGTGGACG
GCGTGGAGGTGCACAATGCTAAGACAAAGCCCAGGGAGGAGCAGTACAATTCCACCTATAGGGTGGTGTCTGTGCTG
ACAGTGCTGCACCAGGATTGGCTGAACGGCAAGGAGTATAAGTGCAAGGTGTCTAATAAGGCCCTGCCTGCTCCAAT
CGAGAAGACCATCAGCAAGGCCAAGGGCCAGCCCAGAGAGCCTCAGGTGTACACACTGCCTCCATCCCGCGACGAGC
TGACCAAGAACCAGGTGTCTCTGACATGTCTGGTGAAGGGCTTCTATCCTTCTGACATCGCTGTGGAGTGGGAGAGC
AATGGCCAGCCAGAGAACAATTACAAGACCACACCCCCTGTGCTGGACAGCGATGGCTCCTTCTTTCTGTATAGCAA
GCTGACCGTGGATAAGTCCCGCTGGCAGCAGGGCAACGTGTTTTCTTGTAGCGTGATGCACGAGGCCCTGCACAATC
ACTACACTCAGAAATCACTGTCACTGTCACCAGGAAAGCATCATCACCATCATCATTGA
```

```
Amino acid sequence
MVKIWVFLFFLSVTTGVHSQVQLVESGGGSVQAGGSLTLSCVASGVTLGRHAIGWFRQAPGKERERVSCIRTFDGIT
SYVESTKGRFTISSNNAMNTVYLQMNSLKPEDTAVYFCALGVTAACSDNPYFWGQGTQVTVSSEPKSSDKTHTSPPS
PAPELLGGSSVFLFPPKPKDTLMISRTPEVTCVVVDVSHEDPEVKFNWYVDGVEVHNAKTKPREEQYNSTYRVVSVL
TVLHQDWLNGKEYKCKVSNKALPAPIEKTISKAKGQPREPQVYTLPPSRDELTKNQVSLTCLVKGFYPSDIAVEWES
NGQPENNYKTTPPVLDSDGSFFLYSKLTVDKSRWQQGNVFSCSVMHEALHNHYTQKSLSLSPGKHHHHHH
```

Key:
Signal peptide
VHH
Hinge
IgG1 Fc (human IgG1 heavy chain constant region)
Histidine tag

b VHH-C5

GenBank accession PX933513

Synthetic construct SARS-CoV-2 spike protein binding camelid VHH-C5 nanobody gene, complete cds

https://www.ncbi.nlm.nih.gov/nuccore/PX933513

```
ATGGTGAAGATTTGGGTGTTCCTGTTCTTTCTGTCTGTGACCACAGGCGTGCACAGCCAGGTGCAGCTGGTGGAGTC
CGGAGGAGGCTCTGTGCAGGCAGGAGGCAGCCTGACCCTGTCCTGCGTGGCCTCTGGAGTGACACTGGGCAGGCACG
CAATCGGATGGTTTAGGCAGGCCCCCGGCAAGGAGAGGGAGAGAGTGTCCTGTATCCGGACCTTCGACGGCATCACA
AGCTACGTGGAGTCCACCAAGGGCAGGTTTACAATCAGCTCCAACAATGCCATGAACACCGTGTACCTGCAGATGAA
CAGCCTGAAGCCCGAGGACACCGCCGTGTATTTCTGCGCACTGGGAGTGACAGCAGCATGTTCTGATAACCCTTATT
TTTGGGGCCAGGGCACCCAGGTGACAGTGTCTAGCCACCACCACCACCACCACTGA
```

```
Amino acid sequence
MVKIWVFLFFLSVTTGVHSQVQLVESGGGSVQAGGSLTLSCVASGVTLGRHAIGWFRQAPGKERERVSCIRTFDGIT
SYVESTKGRFTISSNNAMNTVYLQMNSLKPEDTAVYFCALGVTAACSDNPYFWGQGTQVTVSSHHHHHH
```

c VHH-V

GenBank accession PX933514

Synthetic construct SARS-CoV-2 spike protein binding camelid VHH-V nanobody gene, complete cds

https://www.ncbi.nlm.nih.gov/nuccore/PX933514

```
ATGGTGAAGATTTGGGTGTTCCTGTTCTTTCTGTCTGTGACCACAGGCGTGCACAGCCAGGTGCAGCTGGTGGAGAC
CGGAGGAGGCCTGGTGCAGCCTGGCGGCAGCCTGAGGCTGTCCTGCGCAGCCTCTGGGTTCACCTTCAGCAGCTACG
CAATGGGGTGGGCAAGGCAGGTGCCTGGCAAGGGCCTGGAGTGGGTGAGCTACATCTATTCCGACGGCTCTACCGAG
TACCAGGATTCCGTGAAGGGCCGGTTCACCATCAGCAGAGACAACGCCAAGTCCACAGTGTATCTGCAGATGAACAG
CCTGAAGCCCGAGGATACCGCCGTGTACTATTGTGCCACAGAGGGCTCCCTGGGAGGATGGGGAAGGGACTTTGGCT
CTTGGGGACAGGGCACCCAGGTGACAGTGTCTAGCCACCACCACCACCACCACTGA
```

```
Amino acid sequence
MVKIWVFLFFLSVTTGVHSQVQLVETGGGLVQPGGSLRLSCAASGFTFSSYAMGWARQVPGKGLEWVSYIYSDGSTE
YQDSVKGRFTISRDNAKSTVYLQMNSLKPEDTAVYYCATEGSLGGWGRDFGSWGQGTQVTVSSHHHHHH
```

d VHH-E

GenBank accession PX933515

Synthetic construct SARS-CoV-2 spike protein binding camelid VHH-E nanobody gene, complete cds

https://www.ncbi.nlm.nih.gov/nuccore/PX933515

ATGGTGAAGATTTGGGTGTTCCTGTTCTTTCTGAGCGTGACCACAGGCGTGCACTCCCAGGTGCAGCTGGTGGAGAC
CGGAGGAGGATTTGTGCAGCCTGGCGGCAGCCTGAGGCTGTCCTGCGCAGCCTCTGGCGTGACACTGGACTACTATG
CAATCGGATGGTTCAGGCAGGCCCCTGGCAAGGAGAGGGAGGGCGTGTCCTGTATCGGCAGCTCCGACGGCAGGACC
TACTATTCCGATTCTGTGAAGGGCCGGTTTACCATCTCCAGAGACAACGCCAAGAATACAGTGTACCTGCAGATGAA
CTCTCTGAAGCCCGAGGATACCGCCGTGTACTATTGCGCCCTGACCGTGGGCACATACTATTCTGGCAATTACCACT
ATACATGTAGCGACGATATGGATTATTGGGGCAAGGGCACCCTGGTGACAGTGTCTAGCCACCACCACCACCACCAC
TGA

Amino acid sequence
MVKIWVFLFFLSVTTGVHSQVQLVETGGGFVQPGGSLRLSCAASGVTLDYYAIGWFRQAPGKEREGVSCIGSSDGRT
YYSDSVKGRFTISRDNAKNTVYLQMNSLKPEDTAVYYCALTVGTYYSGNYHYTCSDDMDYWGKGTLVTVSSHHHHHH

Supplementary Figure 2. Recombinant VHHs potently neutralize pseudovirus with SARS-Cov-2 spike protein. Panel **a.** Coomassie-stained SDS-PAGE analysis of recombinant VHH nanobodies specific for the spike protein of SARS-CoV-2 produced in HEK293 cells; sequences from Huo et al 2021[18]. Recombinant VHH (red arrows) and size standards (kDa) are shown on the left. **b.** VHH neutralization titers against pseudotyped lentivirus. Neutralization values ± SEM generated from the pseudotyped LV assay with positive control serum NIBSC (20/130 titer) pseudotyped LV = 0.007 dilution, live virus = 0.005 dilution[20]. IC_{50} mean ± SEM listed for each sample established from log (inhibitor concentration) versus neutralization with variable Hill slope and unrestrained top/bottom values.

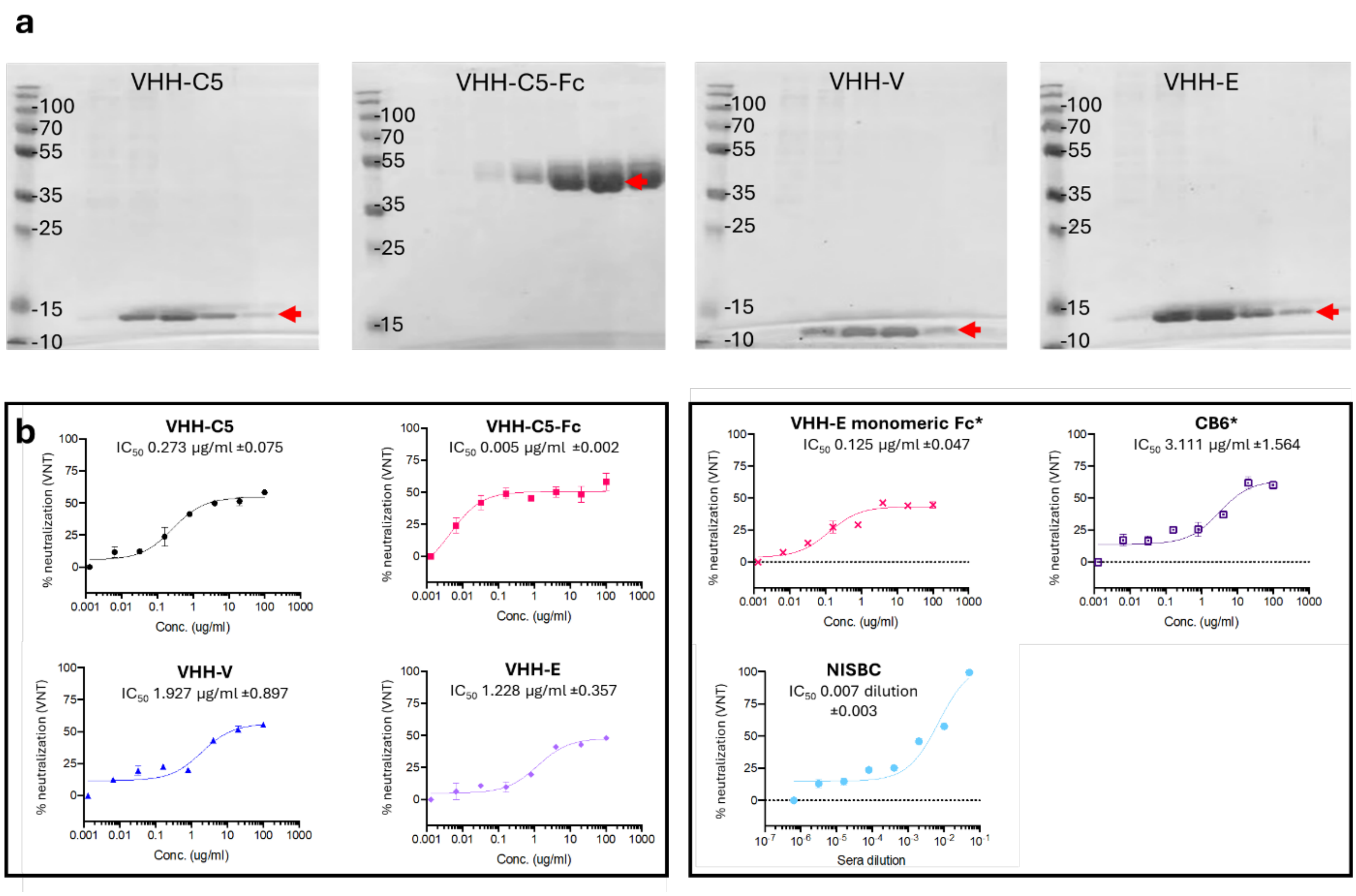

Supplementary Figure 3. FASTA format of nucleotide sequence of the donor transgene coding for C5-Fc antibody in transgenic *Schistosoma mansoni*.

GenBank accession PX933511, Synthetic construct C5-Fc gene, complete cds
https://www.ncbi.nlm.nih.gov/nuccore/PX933511

GAAAATGATATTTCGAGATCAATTATCTGACAATGGAACTCGGTAGATGAcctttccagattgtctccga
ttgatttcacttatctcaatcaccaccagattgtgtcttctcacttggacacgatctgaacggtcctcaattgtgtgcgaacactt
cactttccgatattggctgccattctggtcattgtgaaggtctacgtttttgtggctcataaaaagtcccagctttcggcacaagt
aattgtaagactgccaaactaccaaggaagaggttaaatgacttatctggtaagtgtttttcggaaatattttgctgtatggagtc
actaaccacacacgacctgttttctctactcgagcttgagaaagtcactaacccaagaaaattgaagcctgtaatgttcgtca
tcccttctcttagcgtttatcaagcccacgaatacgtccagccaaaagagaattattgctgccggaatagcggaggtgagtgc
ttctaagtcactgaggttgtcaacatggtcaatgtaaggaaacttctgactgttccttctctcagaatcgaggtagataatatgtg
gaccaagaaatgtgttttgtagacagcgtattttctggtgtgttagcctcgtacataagctaacatcagagattcatgggaaacc
ggtgttgatgttatcactagtgtgaatactagggaatgatctggggtttggaaagtattagttggtcccatcattattcttcacactc
ggagcatcagggcttgatgacttgaaaacgccatcttgtgtctccgtgtaagtggaaatataacataaaataagctttttacgtt
cgagacacatatcgaccagcttagttgggcaatgaatgcaatcttctctttgggattcagtctaggaatttcccatttttaaagg
agaaagttcctttaaaatctttttaagagtctaatttcgagaaactacattaaacagtgtgcgattttatcctcatgtgttcgatga
aggagctagaaaaacattactcggcagtcatccttccgcaaagaaaccatagtgagaggttgttggcgaaaacgtttttgcc
agtaaagatataatattagcagacctgtgactggatgctgatcccacattgcagaataacttgacttctccggacttgaattgt
ccctattaatgaactcatctcctccactatatgtgttctgaaaaattaacctagaaaatattcaaatatcttcaggctataatat
gaagtttcatagtctaagaaaagagattattccatccaaaaccagattttcacgctcaatttcagaaccgtagttcttgtcaac
atttctgatgagtgctagttagtttctgacaaattgaagtgataagaaagtaagaagtgatgtcggaaagaagatgagggtggtg
acaagcagtcgaaatccaggagtcacggctaatccaatggtcgcaccactttgtaagtcggaattcttataaccttcgtggac
agtcgtgttattttcacgctcaattgttgtgaactgggtcttccaatttagcgtctgcagaagtatctgaacgtctcttactcgctta
agtcagagacaaactagtgagacattttcaaggtcttcacaattgttttctgttgatttagatgtgaaaaatcattcatatgatgc
acttggagatgtttaagttccgatctaacgaagcatatctccaacagttaactgctctaataaattgatgacttcacgtgaacta
aaacccataaagataaaatggcaaactttattttacattattacctaaacttttgtcatcttgaaactcgaaaaatgtggcactt
gaatgagaagttgctgactaacaaaaacgtttcataatgaattaggaggttcagactgtaacaagttattttctgaaagtaaat
gtgactagaatgtggtttgagtaagtaacaatcacctcgacgaataccttgttttactgccctgaattgaaggtagcgctaaata
aacgtagtatagactatatatagccagcaaaatcgaaaggctgccatacttgttcgttagtgtaaacgatagaccttacgCA
CCATGGTGAAATTAGTGCTGGTGCCCCTGCTGGCCCTGCTGGCCGTGTCCGTGCACCAAGT
GCAGCTGGTCGAGAGCGGAGGCGGCTCTGTGCAGGCCGGCGGATCTCTGACCCTGTCTTG
CGTGGCCAGCGGCGTGACCCTGGGAAGGCACGCCATCGGATGGTTCCGGCAGGCTCCTG
GCAAAGAGCGGGAAAGAGTGTCCTGCATCCGGACCTTCGACGGCATCACAAGCTACGTGGA
AAGCACCAAGGGCAGATTCACCATCAGCAGCAACAACGCTATGAACACCGTGTACCTGCAG
ATGAATTCCCTGAAGCCAGAGGATACCGCCGTGTACTTCTGCGCCCTCGGCGTGACAGCCG
CCTGCAGCGACAACCCCTACTTTTGGGGCCAGGGAACCCAGGTGACCGTGTCCAGCGAGC
CTAAAAGCAGCGATAAGACCCACACCAGCCCACCTTCTCCTGCTCCTGAGCTGCTGGGCGG
CTCTTCTGTGTTTCTGTTCCCCCCTAAGCCCAAGGACACACTCATGATCAGCAGAACCCCTGA
GGTGACCTGCGTGGTCGTGGACGTGAGCCACGAAGATCCTGAAGTGAAATTCAACTGGTATG
TGGACGGCGTCGAGGTGCACAACGCTAAAACAAAGCCTAGAGAGGAACAGTACAACAGCAC
ATACAGAGTGGTGTCCGTCCTGACCGTGCTGCACCAAGACTGGCTGAACGGCAAGGAATAC

AAGTGCAAGGTGTCTAACAAGGCCCTGCCCGCCCCTATCGAGAAGACAATTAGCAAGGCCAAGGGCCAACCTAGAGAACCCCAGGTGTATACACTGCCTCCAAGCCGGGACGAGCTGACCAAGAACCAGGTTAGCCTGACATGTCTGGTGAAGGGCTTCTACCCCTCCGACATCGCCGTGGAATGGGAGAGCAACGGCCAGCCTGAGAACAACTACAAGACCACCCCTCCTGTGCTGGACAGCGATGGCAGCTTTTTCCTGTACAGCAAGCTGACAGTGGATAAGTCCAGATGGCAGCAGGGCAATGTTTTCAGCTGTTCTGTGATGCACGAGGCCCTGCATAATCACTACACCCAGAAAAGCCTGAGCCTGAGCCCCGGCAAGCACCATCATCACCATCAT*TAA*tggaggttaactttaattttgttcatttaacttaataaacgtttaatttaacttattaaaatgttttcttcctaatttgtagcgtatcttagtgtaactgtgcttcctattagattgtaattctatcgtaatctgcaattaggctcatggggatatgttatgccaattttccgaaaattgttagcacttaggtttcggtgtttttttactaggatgtcatgttttattcatatttgcgtccttgctcttgtgtgtggtatgcgtcgctatggtctcagctttgggctgcattgttaagtttgagATTGGGATGGTGTATAGATGTGCATTAGTCACAGACTACTATAAGAAGA

Key:

Homology arm

Promoter region

Kozak sequence

Signal peptide, underlined

Coding region C5 VHH – IgG1 Fc

Histidine tag

Terminator region

Supplementary Figure 4. Collage of several informative outcomes observed during establishment of the C5-Fc line of transgenic *S. mansoni*, dealing with the P0, F1 to F2 generations.

Panel **a.** Representative micrographs – bright field at left, fluorescence at right – of *in vitro*-laid eggs of *Schistosoma mansoni* following transfection by electroporation with ATTO 550-labeled RNPs and the donor transgene. Images revealed that RNP materials entered the egg and were in and/or around the developing embryo, indicating that RNPs reached vicinity of the germ tissue (top and middle panel). Bottom panel, example from control group eggs transfected only with the donor transgene only (no RNPs). **b.** Summary of numbers of snails shedding transgenic cercariae. The presence of the C5-Fc transgene was tested in batches of 10s to 100s of cercariae as they exited the snail. When established that a snail was shedding transgenic cercariae, additional cercariae shed from the same snail were used to infect mice. **c.** liver of naïve (non-infected mouse); **d.** liver of mouse infected for >80 days with P0 generation male and female cercariae of the C5-Fc *S. mansoni* line. **e.** Confirmation of the presence of the C5-Fc transgene. Representative agarose gel showing PCR amplification of the transgene (TG) from genomic DNA isolated from F1 female and from mixed sex adult worms recovered from TG cercariae-infected mice. The donor only lanes C-F1 and C-F2 (donor-only controls) represent the products from the PCR of TG from the worms from mice infected with donor transfected-eggs to miracidium to cercariae. The amplicon of ~4,004 bp (i.e., the expected size) confirmed the guide RNA programmed Cas9 knockin of the C5-Fc donor transgene at the targeted genome safe harbor in the *Schistosoma mansoni*. Size standards in base pairs (bp) shown at left.

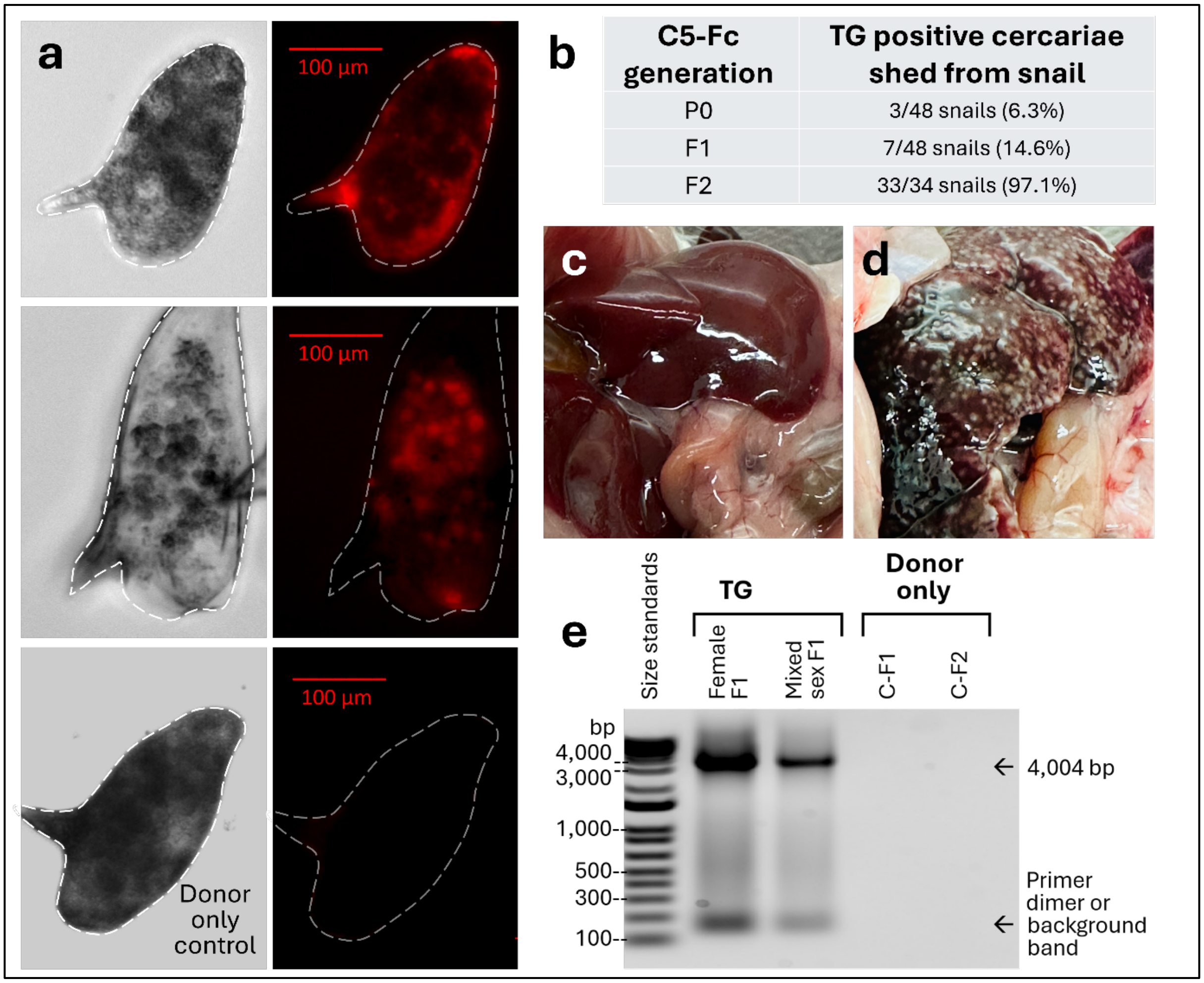
a
100 µm
100 µm
100 µm
Donor
only
control
b
C5-Fc generation
TG positive cercariae shed from snail
P0
3/48 snails (6.3%)
F1
7/48 snails (14.6%)
F2
33/34 snails (97.1%)
c
d
e
Size standards
TG
Female F1
Mixed sex F1
Donor only
C-F1
C-F2
bp
4,000
3,000
1,000
500
300
100
4,004 bp
Primer dimer or background band

Supplementary Figure 5. Representative observations of C5-Fc transgenic *Schistosoma mansoni* across generations through F9.

Panel **a.** Photographs of mouse livers resected at necropsy 62 days after infection with 105 cercariae of the F6 generation of the transgenic line, showing hepatic granuloma. **b.** PCR confirmation of C5-Fc transgene in genomic DNA from F5-generation transgenic C5-Fc schistosomes. Representative agarose gel stained with SYBR SAFE showing PCR products generated with transgene-specific primers C5VHH-F and FcIgG1-R. Lanes: 1, DNA size marker; 2, primers only (no template control); 3, C5-Fc F5 gene genomic DNA (gDNA), no primers; 4, wild-type (negative control) genomic DNA from the NMRI strain of *S. mansoni* (no band at expected transgene size); 5–6, gDNA from two independent pools of F5-generation transgenic adult, mixed sex *S. mansoni* worms, showing the expected C5-Fc amplicon of 302 nt (blue arrow); 7, empty lane; 8,. Positive control – primers, 70 pg donor transgene plasmid (positive control), showing the expected C5-Fc amplicon of 302 nt (blue arrow). PCR conditions: [primers C5VHH-F and FcIgG1-R], annealing temperature 55 °C, 35 cycles. Gel: 1% agarose, 96 volts for 30 minutes. The presence of the 302 bp C5-Fc amplicon in two independent F5 transgenic gDNA pools (lanes 5–6) and the plasmid positive control, with no band in wild-type or no-template controls, confirmed stable genomic integration of the C5-Fc transgene in the F5 generation. **c.** Pairs of F6 generation adult worms *in copula,* perfused from the portal circulation of mice. Length of worms, ~ 10 mm each. **d.** End-point RT-PCR detection of C5-Fc transgene and GAPDH in adult *Schistosoma mansoni* cDNA. Lane 1, SYBR SAFE–stained 1% agarose gel of RT-PCR products run alongside a 1 kb Plus DNA ladder (Invitrogen); 2, total cDNA/RNA from adult *S. mansoni* and transgene primers C5VHH-F and FcIgG1-R, with a clear band visible at 302 bp corresponding to the C5-Fc transgene; 3, primers without cDNA; 4, lane is empty; 5, total cDNA/RNA from adult *S. mansoni* and *Smgapdh* primers C5VHH-F and FcIgG1-R, with a clear band visible at 302 bp corresponding to the C5-Fc transgene, and the strong band at 285 bp corresponded to the reference gene *Smgapdh*. 6, 1 kb Plus DNA ladder; 7 lane is empty; 8, positive control, 70 pg donor transgene plasmid (positive control), transgene primers, and displays the expected 302 bp band. Bands were visualized with SYBR SAFE. Amplificon of the 302 bp fragment of the C5-Fc transgene in adult *S. mansoni* cDNA with robust *Sm*GAPDH (285 bp) and the absence of any product in the no-template control confirmed specific transgene transcription, validated by the plasmid positive control. **e.** Photographs documenting hatching of C5-Fc F9 generation eggs and swimming of miracidia in water in a beaker (left and right sides show low and higher magnifications, respectively. **f.** Western blot of proteins separated on a 4–1% gradient SDS–PAGE and probed with HRP-conjugated rabbit IgG monoclonal anti–6×His tag (1:5,000). Lane 1: prestained protein standards (Sea Blue Plus2, Fisher Scientific). Lanes 2–4: individual mouse sera (1.5 µl per lane) from three separate mice infected with F8-generation C5–

Fc transgenic *S. mansoni* (one mouse per lane), sampled at day 70 after infection with 135 cercariae. Lanes 5–7: individual mouse sera (1.5 μL per lane) sampled at day 50 after infection with.180 cercariae of wild-type *S. mansoni* (one mouse per lane). Lanes 8–10: recombinant C5–Fc produced in HEK-293 cells (2 ng in lane 8; 10 ng in lane 9 and 20 ng in lane 10). Molecular mass markers indicated at left; expected position of C5–Fc is marked with a blue arrow in the C5-Fc mouse sera and black arrow in recombinant C5-Fc lanes. The presence of the C5–Fc signal in mouse sera confirmed continued inheritance and functional expression of the C5–Fc transgene through at least eight filial generations. The PCR based assays shown in panels b and d were repeated twice, with similar results both times, and the western in panel f was repeated three times, with sera from the same mice, with the same outcome for each replicate.

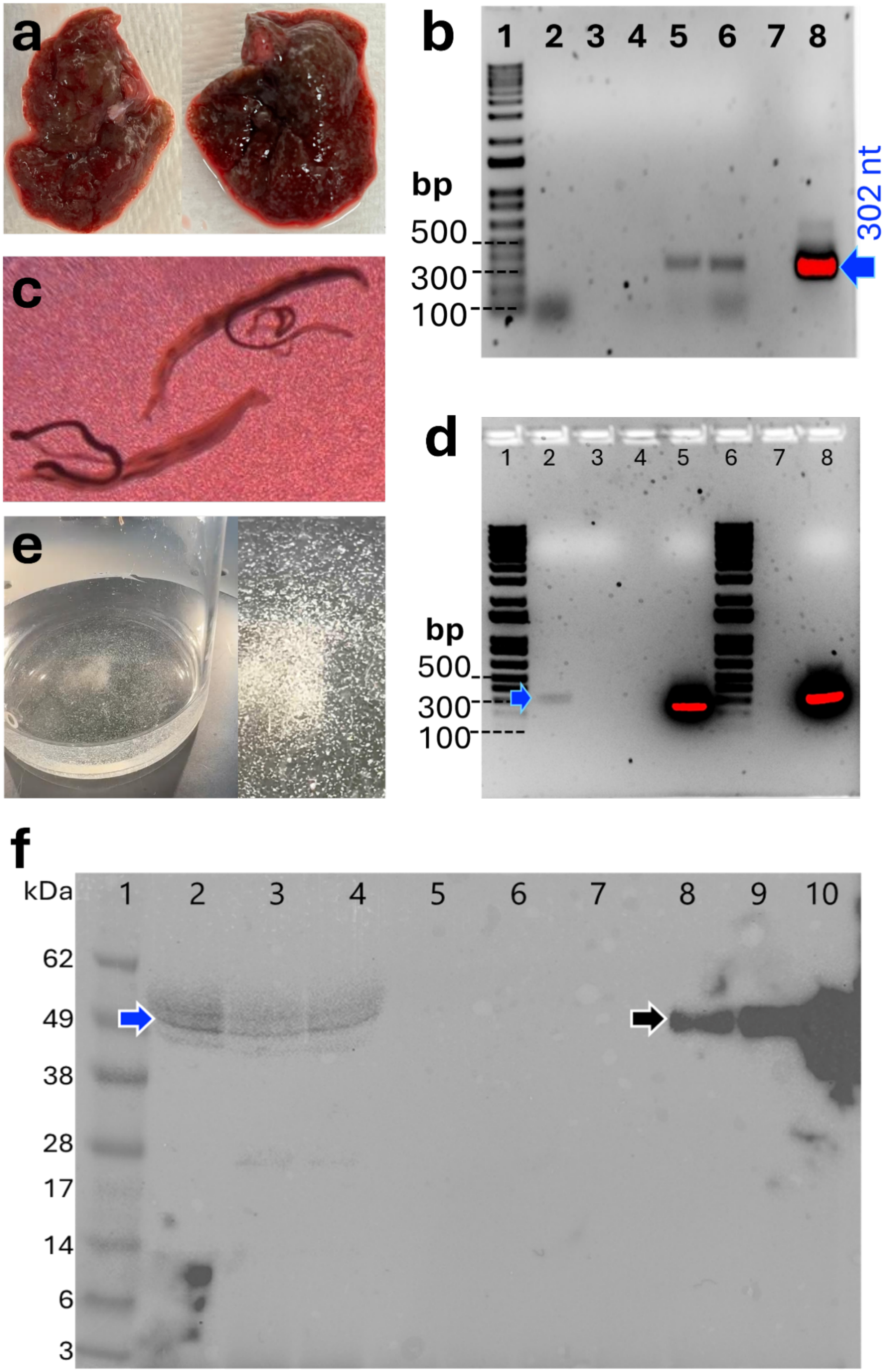
a
b
1 2 3 4 5 6 7 8
bp
500
300
100
302 nt
c
d
1 2 3 4 5 6 7 8
bp
500
300
100
e
f
kDa
1 2 3 4 5 6 7 8 9 10
62
49
38
28
17
14
6
3

Supplementary Movie 1. Hatching of *Schistosoma mansoni* eggs of F9 generation of the C5-Fc line, with the released miracidia actively in a beaker of pond water. The video was recorded on an iPhone 16 showing a beaker containing pond water with schistosome eggs recovered from the livers of mice experimentally infected with F8 generation C5-Fc schistosomes. Miracidia hatched from the eggs, which were on the bottom of the beaker, actively swimming in water. Playback speed is real time (1×); estimated length of miracidium, ~120 µm (based on egg length). Brightfield illumination; no staining. File format: MP4; duration 00:00:25; resolution 1920×1080.

Supplementary Movie 2. *Biomphalaria glabrata* snail during exposure to infection with miracidia of the F9 generation of transgenic *S. mansoni*. Continuous video recorded on an iPhone 16 showing a single *B. glabrata* adult (shell diameter ~5 mm) following the addition of recently hatched *S. mansoni* miracidia to the observation field; ~100 miracidia dispensed into a beaker holding 10 snails in 10 ml pond water. Following miracidia addition, snails exhibited movement of tentacles and partial withdrawal into the shell, episodic body extension, and locomotory changes. Lighting and focus reflect handheld mobile phone video captured in standard laboratory lighting.

Supplementary Table 1. Oligonucleotide primers and guide RNAs

Oligonucleotide	Sequence, 5′ to 3′	Expected amplicon (bp)
5′Out-F	TGTTATCGTCCGTCGCTTCA	4,004
5′Out-R	TAGTCACTTGCAGTGGTGCAT	
C5VHH-F	AAGCTACGTGGAAAGCACCA	302
FcIgG1-R	TCATGAGTGTGTCCTTGGGC	
Smp_011570.1F	TGGATGTTGGATAAGCTGGG	404
Smp_011570.1R	TGGATCTGGATATCGAATGGTC	
Sm-W6-F	TGTGAAGCAAAGTGTTCACTG	195
Sm-W6-R	TTCATCAAGTCAATCACAGCTC	
5AmMC6-50ntGSH1-UbiPro-F	5AmMC6/ATGCCACAAGTGTTTACAAGGAAAATGATATCCC GAGATCAATTATCTGACCTTTCCAGATTGTCTCCGATTG	3,598
5AmMC6-50ntGSH1-UbishTer-R	5AmMC6/ACACCATCCCAATTCCAGCTATTGAACCAGCCTTC ATCTACCGAGTTCCACTCAAACTTAACAATGCAGCCC	
*Sm*GAPDH-F	ATGGGACATTTCCAGGCGAG	285
*Sm*GAPDH-R	CCAACAACGAACATGGGTGC	
Guide RNA 1	GGAACTCGGTAGATGAAGGC(TGG)	NA[1]
Guide RNA 2	ATGAAGGCTGGTTCAATAGC(TGG)	NA
Guide RNA 3	CGTGGTTCAATAGCTGGAAT(TGG).	NA

[1]. Not applicable
