# Supplementary material for: Heritable transgenic schistosomes as a living platform for SARS-CoV-2 neutralizing antibody secretion
Source: Nat Commun. 2026 Aug 26;17:9052. doi: 10.1038/s41467-026-76465-9 (PMC13518888; doi:10.1038/s41467-026-76465-9)
Supplement: Supplementary file 2 [file 41467_2026_76465_MOESM5_ESM.pdf]

nature portfolio

Corresponding author(s): Paul J. Brindley, Alex Loukas

Last updated by author(s): 2026-July-06

Reporting Summary

Nature Portfolio wishes to improve the reproducibility of the work that we publish. This form provides structure for consistency and transparency in reporting. For further information on Nature Portfolio policies, see our Editorial Policies and the Editorial Policy Checklist.

Statistics

For all statistical analyses, confirm that the following items are present in the figure legend, table legend, main text, or Methods section.

n/a	Confirmed	
☐	☒	The exact sample size (n) for each experimental group/condition, given as a discrete number and unit of measurement
☐	☒	A statement on whether measurements were taken from distinct samples or whether the same sample was measured repeatedly
☐	☒	The statistical test(s) used AND whether they are one- or two-sided<br>*Only common tests should be described solely by name; describe more complex techniques in the Methods section.*
☐	☒	A description of all covariates tested
☐	☒	A description of any assumptions or corrections, such as tests of normality and adjustment for multiple comparisons
☐	☒	A full description of the statistical parameters including central tendency (e.g. means) or other basic estimates (e.g. regression coefficient) AND variation (e.g. standard deviation) or associated estimates of uncertainty (e.g. confidence intervals)
☐	☒	For null hypothesis testing, the test statistic (e.g. F, t, r) with confidence intervals, effect sizes, degrees of freedom and P value noted<br>*Give P values as exact values whenever suitable.*
☒	☐	For Bayesian analysis, information on the choice of priors and Markov chain Monte Carlo settings
☐	☒	For hierarchical and complex designs, identification of the appropriate level for tests and full reporting of outcomes
☒	☐	Estimates of effect sizes (e.g. Cohen's d, Pearson's r), indicating how they were calculated

Our web collection on statistics for biologists contains articles on many of the points above.

Software and code

Policy information about availability of computer code

Data collection	SnapGene v6.0 to v8.2
Data analysis	SnapGene v6.0 to v8.2<br><br>GraphPad Prism v11.02

For manuscripts utilizing custom algorithms or software that are central to the research but not yet described in published literature, software must be made available to editors and reviewers. We strongly encourage code deposition in a community repository (e.g. GitHub). See the Nature Portfolio guidelines for submitting code & software for further information.

Data

Policy information about availability of data

All manuscripts must include a data availability statement. This statement should provide the following information, where applicable:

- Accession codes, unique identifiers, or web links for publicly available datasets
- A description of any restrictions on data availability
- For clinical datasets or third party data, please ensure that the statement adheres to our policy

Data and materials availability
The nucleotide sequence data generated in this study have been deposited in the GenBank, specifically the nucleotide sequence of the donor transgene has been assigned GenBank accession PX933511 and the nucleotide sequences of four VHHs expressed in mammalian cells are assigned PX933512–PX933515.
PX933511, https://www.ncbi.nlm.nih.gov/nuccore/PX933511
PX933512, https://www.ncbi.nlm.nih.gov/nuccore/PX933512.1/
PX933513, https://www.ncbi.nlm.nih.gov/nuccore/PX933513
PX933514, https://www.ncbi.nlm.nih.gov/nuccore/PX933514
PX933515, https://www.ncbi.nlm.nih.gov/nuccore/PX933515

Other accessions cited:

AF089728.1, https://www.ncbi.nlm.nih.gov/nuccore/AF089728.1/
RCN53414.1, https://www.ncbi.nlm.nih.gov/protein/RCN53414.1
HQ880215, https://www.ncbi.nlm.nih.gov/nuccore/HQ880215
Smp_011570.1, https://www.ncbi.nlm.nih.gov/nuccore/?term=Smp_011570.1
MN908947, https://www.ncbi.nlm.nih.gov/nuccore/MN908947

All other data, including source data for GraphPad Prism–based statistical analyses, curve fitting, and graphical representations, are available in the main text and/or the Supporting Information and/or the Source Data file.

Research involving human participants, their data, or biological material

Policy information about studies with human participants or human data. See also policy information about sex, gender (identity/presentation), and sexual orientation and race, ethnicity and racism.

Reporting on sex and gender	*Use the terms sex (biological attribute) and gender (shaped by social and cultural circumstances) carefully in order to avoid confusing both terms. Indicate if findings apply to only one sex or gender; describe whether sex and gender were considered in study design; whether sex and/or gender was determined based on self-reporting or assigned and methods used. Provide in the source data disaggregated sex and gender data, where this information has been collected, and if consent has been obtained for sharing of individual-level data; provide overall numbers in this Reporting Summary. Please state if this information has not been collected. Report sex- and gender-based analyses where performed, justify reasons for lack of sex- and gender-based analysis.*
Reporting on race, ethnicity, or other socially relevant groupings	*Please specify the socially constructed or socially relevant categorization variable(s) used in your manuscript and explain why they were used. Please note that such variables should not be used as proxies for other socially constructed/relevant variables (for example, race or ethnicity should not be used as a proxy for socioeconomic status). Provide clear definitions of the relevant terms used, how they were provided (by the participants/respondents, the researchers, or third parties), and the method(s) used to classify people into the different categories (e.g. self-report, census or administrative data, social media data, etc.) Please provide details about how you controlled for confounding variables in your analyses.*
Population characteristics	*Describe the covariate-relevant population characteristics of the human research participants (e.g. age, genotypic information, past and current diagnosis and treatment categories). If you filled out the behavioural & social sciences study design questions and have nothing to add here, write "See above."*
Recruitment	*Describe how participants were recruited. Outline any potential self-selection bias or other biases that may be present and how these are likely to impact results.*
Ethics oversight	*Identify the organization(s) that approved the study protocol.*

Note that full information on the approval of the study protocol must also be provided in the manuscript.

Field-specific reporting

Please select the one below that is the best fit for your research. If you are not sure, read the appropriate sections before making your selection.

☒ Life sciences ☐ Behavioural & social sciences ☐ Ecological, evolutionary & environmental sciences

For a reference copy of the document with all sections, see nature.com/documents/nr-reporting-summary-flat.pdf

Life sciences study design

All studies must disclose on these points even when the disclosure is negative.

Sample size	Sample sizes were constrained by the availability of transgenic schistosome material during the establishment of the transgenic line, as the number of mice infected with transgenic schistosomes in each generation was limited by the number of available schistosome larvae.
Data exclusions	Data were not excluded.
Replication	Replicates were in employed in salient assays, including 1) ELISA to assess the concentration of C5-Fc VHH biological therapeutic released from schistosomes; 2) in PCR analysis for the presence of the C5-Fc transgene in the schistosome genome; 3) for western blot analysis of the presence of the C5-Fc biologic in blood (sera) of mice infected with the transgenic worms; and 4) in PRNTs (plaque reduction neutralization test) of the neutralization capacity of mouse sera to block the SARS-Co-V-2 virus from infecting Vero cells.
Randomization	Randomization was not relevant to this investigation. The goal was to derive a heritable transgenic line of schistosomes, and wherein the transgenic line would secrete a mammalian antibody. Accordingly, the focus on the study was on the transgenic schistosomes.
Blinding	The pseudoptyped lentivirus assays were carried out in the University of Queensland (UQ) using recombinant VHHs provided by the James Cook University lab. In this sense the assay was blinded because the UQ lab was not yet privy by plans from the other labs including the lead lab at George Washington University(GWU) to include a VHH sequence as a transgene in the schistosome genome. The Plaque Reduction Neutralization Test (PRNT) of mouse sera and worm secretions was carried at the University of Nebraska Medical Center (UNMC) lab, with samples from GWU. GWW sent the samples in coded format and UNMC did not have the code at the time the PRNT assay was performed.

Reporting for specific materials, systems and methods

We require information from authors about some types of materials, experimental systems and methods used in many studies. Here, indicate whether each material, system or method listed is relevant to your study. If you are not sure if a list item applies to your research, read the appropriate section before selecting a response.

Materials & experimental systems

n/a	Involved in the study	
☐	☒	Antibodies
☐	☒	Eukaryotic cell lines
☒	☐	Palaeontology and archaeology
☐	☒	Animals and other organisms
☒	☐	Clinical data
☒	☐	Dual use research of concern
☒	☐	Plants

Methods

n/a	Involved in the study	
☒	☐	ChIP-seq
☒	☐	Flow cytometry
☒	☐	MRI-based neuroimaging

Antibodies

Antibodies used	For “ELISA for detection of C5-Fc nanobody secreted by transgenic worms”, “...rabbit anti-camelid (camel and llama) VHH-conjugated to horse radish peroxidase [HRP] cocktail used at 1: 4,000 dilution (MonoRab, GenScript catalog A02016).”<br>For “Western blot”, “...an HRP-conjugated rabbit polyclonal antibody detecting 6X His tag (Abcam, catalog no. ab1187), diluted 1: 5,000 in TBST with 1% nonfat dry milk...”
Validation	Both these antibodies were purchased from commercial vendors.

Eukaryotic cell lines

Policy information about cell lines and Sex and Gender in Research

Cell line source(s)	Expi293F HEK (human embryonic kidney) 293 cells (cat# A14527, Thermo Fisher Scientific). Verified mycoplasma free and STR identity confirmed before purchase.<br><br>HEK293T cells used in the lentivirus plaque assay were purchased from XXX and certified as mycoplasma-free at the time of purchase. HEK293T is not listed on ICLAC v14 list. Sigma-Aldrich, Millipore Sigma, St. Louis, MO, catalog no. 12022001-1VL.<br><br>Vero cells (green monkey cells) obtained from the ATCC (American Type Culture Collection), and STR typed by ATCC and certified as mycoplasma-free at th time of purchase. Vero E6 is not listed on ICLAC v14 list.

Authentication	Expi293F HEK (human embryonic kidney) 293 cells (cat# A14527, Thermo Fisher Scientific). Verified mycoplasma free and STR identity confirmed before purchase.<br><br>HEK293 T: Verified mycoplasma free and STR identity confirmed before purchase(Millipore Sigma/Sigma-Aldrich, catalog no. 12022001-1VL<br><br>Vero E6 cells, Vero E6 cells (ATCC catalog no. CRL-1586). Verified mycoplasma free and STR identity confirmed before purchase.
Mycoplasma contamination	Expi293F HEKVero E6, and HEK293T - not contaminated.
Commonly misidentified lines<br>(See ICLAC register)	*Name any commonly misidentified cell lines used in the study and provide a rationale for their use.*

Animals and other research organisms

Policy information about studies involving animals; ARRIVE guidelines recommended for reporting animal research, and Sex and Gender in Research

Laboratory animals	Mouse (Mus musculus)
Wild animals	*Provide details on animals observed in or captured in the field; report species and age where possible. Describe how animals were caught and transported and what happened to captive animals after the study (if killed, explain why and describe method; if released, say where and when) OR state that the study did not involve wild animals.*
Reporting on sex	With regard to sex as a biological variable, we used only female mice as the schistosome host, reasoning that the establishment of the transgenic schistosome line in one host sex provided more control because it removed sources of biological variability including sex-based diffrences in immune response, hormone levels, parasite burden and pathology.
Field-collected samples	*For laboratory work with field-collected samples, describe all relevant parameters such as housing, maintenance, temperature, photoperiod and end-of-experiment protocol OR state that the study did not involve samples collected from the field.*
Ethics oversight	Mice (female, Swiss Webster, 8 wk old) infected with S. mansoni (NMRI strain; origin Puerto Rico72) were obtained from the Schistosomiasis Resource Center (Biomedical Research Institute, Rockville, MD) within seven days of infection by cercariae (180 cercariae/mouse/percutaneous infection). The mice were housed at the Animal Research Facility of George Washington University, which is accredited by the American Association for Accreditation of Laboratory Animal Care (AAALAC no. 000347) and has the Animal Welfare Assurance on file with the National Institutes of Health, Office of Laboratory Animal Welfare, OLAW assurance A3205, using a 12 hour dark/ 12 hour light cycle, 23°C ambient temperature, and 60% humidity. All procedures employed were consistent with the Guide for the Care and Use of Laboratory Animals. The Institutional Animal Care and Use Committee of the George Washington University approved the protocol used for maintenance of mice and recovery of schistosomes, protocol numbers A2020-037 and A2034-074, as did the Animal Care and Use Review Office of the US Army Medical Research and Development Command, ACURO protocol number NIWC-8735.e003.

Note that full information on the approval of the study protocol must also be provided in the manuscript.

Plants

Seed stocks	*Report on the source of all seed stocks or other plant material used. If applicable, state the seed stock centre and catalogue number. If plant specimens were collected from the field, describe the collection location, date and sampling procedures.*
Novel plant genotypes	*Describe the methods by which all novel plant genotypes were produced. This includes those generated by transgenic approaches, gene editing, chemical/radiation-based mutagenesis and hybridization. For transgenic lines, describe the transformation method, the number of independent lines analyzed and the generation upon which experiments were performed. For gene-edited lines, describe the editor used, the endogenous sequence targeted for editing, the targeting guide RNA sequence (if applicable) and how the editor was applied.*
Authentication	*Describe any authentication procedures for each seed stock used or novel genotype generated. Describe any experiments used to assess the effect of a mutation and, where applicable, how potential secondary effects (e.g. second site T-DNA insertions, mosiacism, off-target gene editing) were examined.*
