# Supplementary material for: Heritable transgenic schistosomes as a living platform for SARS-CoV-2 neutralizing antibody secretion
Source: Nat Commun. 2026 Aug 26;17:9052. doi: 10.1038/s41467-026-76465-9 (PMC13518888; doi:10.1038/s41467-026-76465-9)
Supplement: Supplementary file 3 [file 41467_2026_76465_MOESM6_ESM.pdf]

nature portfolio

Peer Review File

Heritable transgenic schistosomes as a living platform for SARS CoV 2 neutralizing antibody secretion

Corresponding Author: Professor Paul Brindley

This manuscript has been previously reviewed at another journal. This document only contains information relating to versions considered at Nature Communications.

This file contains all reviewer reports in order by version, followed by all author rebuttals in order by version.

Version 0:

Reviewer comments:

Reviewer #1

(Remarks to the Author)
This manuscript by Ittiprasert reports some groundbreaking work in functional helminth genomics. The field of helminthology has long been plagued by the difficulty of successful and effective transgenics. While progress has been made in nematodes, this
generation and propagation of a heritable transgenic schistosome line is certainly a breakthrough.

Additionally, the authors have utilized this approach of a transgenic parasite that lives in the blood to be an in vivo "living foundry" to continuously secrete a therapeutic neutralizing antibody within a mammalian host directly into the blood system. It is truly remarkable that they managed to get this to work. The manuscript is also well-written, the experimental data and design appear solid, and this work will likely have a high impact on the field.

Some of the strengths of their strategy is to deploy multiplexed CRISPR/Cas9 homology-directed knock-in targeting a predicted genome safe harbor (GSH1) in immature eggs. Propagating this line through snail and mouse hosts up to the F2 generation—where 97% of snails shed transgenic cercariae—is a remarkable technical achievement.

Interestingly, the authors decided to make the parasites express transgenic antibodies by fusing the anti-SARS-CoV-2 C5 VHH to a human IgG1 Fc domain. Driving expression with an endogenous, ubiquitously expressed schistosome ubiquitin promoter led to constitutive production without epigenetic silencing. They could demonstrate the functional utility of these transgenic anitbodies to neutralize live SARS-CoV-2.

My only critique would be that the authors need to have a more nuanced perspective regarding clinical safety and translation.
While Male-Only Infections have been used in controlled human infections, study participants still reported signs of Katayama fever, which acts earlier through an independent mechanism from the eggs that cause pathology in the liver. This systemic hypersensitivity reaction to migrating schistosomula and maturing adults can cause significant morbidity, including fever, fatigue, myalgia, and eosinophilia. Hence the authors need to discuss the pathological risks of Katayama fever associated with male-only infections in the Discussion. Acknowledging this downside will provide a more balanced, comprehensive, and realistic view of the clinical hurdles facing the deployment of schistosomes as living foundries in humans.

Reviewer #2

(Remarks to the Author)

The manuscript describes generation of stable transgenic lines of Schistosoma using CRISPR/Cas9 knock-in, and demonstrates that expression of an engineered antibody targeting the spike protein of SARS-CoV-2 from such transgene results in a functional antibody against SARS-CoV-2. This is a very significant technical advance, since stable and heritable transgenesis in Schistosoma will be instrumental for further investigations of this parasitic flatworm but also because it

opens a venue for using this animal as a vehicle for delivery of therapeutic proteins.

The work is clearly described and technically solid. I'd like to congratulate the authors!

I have only several comments:

Lines 67-68: "To date, heritably transgenic lines of schistosomes or indeed any platyhelminth –parasitic or free living - have not been described." – This is not correct and disappointing that the authors are not sufficiently informed about broader platyhelminth literature. Stable transgenesis in a free-living flatworm Macrostomum was described in 2017 (also in Nature Communications), and several follow-up papers with more transgenic lines have been published since.

Lines 258-260 – primer sequences not needed here but should be in the table with all primers.

The efficiency of CRISPR/Cas9 knockin is not discussed. The numbers are mentioned in the text but a small table will be very useful. If I understand correctly, the numbers are based on a single experiment. Do the authors have more information about the reproducibility of knock-ins?

Some very specific choices were made in the knock-in protocol, e.g. the use of oligos modified in a certain way. A discussion about these particular choices and potential alternatives that might further increase the efficiency will be useful.

Reviewer #3

(Remarks to the Author)
Summary
The authors of this paper demonstrate 1) stable introduction of a new gene that is now heritable into a platyhelminth, and 2) ability to utilize schistosomes as vehicles to produce novel
proteins for long periods of time in an infected host. The authors used CRISPR/Cas9 technology to knock-in a transgene construct "C5-Fc" encoding an anti-SARS-COV-2 spike VHH fused to the human IgG1 Fc domain. They utilized three overlapping guide RNAs for the CRISPR/Cas9 knock-in and included the S. mansoni ubiquitin promoter and a secretion signal peptide from the hookworm protein ASP2. The resultant C5 Fcs dimerized, producing a bivalent antibody against SARS-CoV-2. Transfection was conducted via electroporation of the three guide RNAs along with the C5-Fc geneinto immature schistosome eggs. Miracidia hatched from these eggs were then used to infect snails, and then cercariae from the snail used to infect mice. Mice were infected sequentially with male cercariae and then, a few weeks later, with female cercariae. Eggs obtained from the murine infection were then used to generate F1 generation miracidia to infect snails, from which cercariae were derived to infect a 2nd generation of mice. The authors show that both excretory/secretory product from transgenic flukes, as well as sera from infected mice, have C5-Fc (by ELISA) which can effectively neutralize SARS-CoV-2 (shown via both lentiviral pseudovirus assay as well as plaque reduction assay using live virus.

Overall review
Overall, the paper is quite remarkable, as it is the first to my knowledge to demonstrate stable introduction of a new, heritable gene into a platyhelminth. Additionally, the utilization of Schistosomes as a potential long-lived factory for production of a specific protein in an infected host is novel and may have important translational implications (if not with Schistosomes, then perhaps with a less pathogenic long-lived helminth).
The paper is well written, the scientific approach appears robust, and the figures, especially the one on assembly of the transgene, are very clear and easy to follow. The methods are also exceptionally well written, with sufficient detail to enable replication of the study.

Major critiques: none

Minor critiques:
1) In Figure 5, when using sera of infected mice, the authors should specify whether the sera was from mice infected with the first round of cercariae (F1) or the 2nd round (F2).
2) If the data from the serum studies in Figure 5 is from the F1 generation, did the authors conduct studies to confirm that the F2 continued to produce C5-Fc in vivo? This may have been mentioned somewhere in the text but I didn't catch it.

Reviewer #4

(Remarks to the Author)
This is a remarkable study demonstrating the generation of transgenic schistosoma lines that secrete neutralizing nanobodies to SARS-CoV2. Together, the authors combine CRISPR/Cas, nanobody design, and Schistosoma manipulation for a molecular tour-de-force in generating heritable parasites that secrete a functional therapeutic protein against SARS-CoV2. Prior studies have reported the generation of transgenic helminths, but this work is the first of its kind in generating heritably transgenic schistosoma lines that produce a therapeutic protein. Based on this novelty, this study will be of high significance to many fields of research, from therapeutic technologies to parasitology and immunology.
The study provides thorough detail and rational for the design and validation of the transgenic parasites, and reports several findings: 1/ the successful generation of transgenic schistosoma that produce the camelid antibody against SARS-CoV2,

specifically VHH-C5-Fc that was the most potent in plaque assays, 2/ the successful infection of mice with single or mixed sexes that resulted in long-term secretion (24 weeks) of the functional nanobody in the mouse blood, 3/the strong neutralization potential for the nanobodies from the infected mouse serum.
Overall, this is a well-written study with convincing results and provides the research field with a new therapeutic delivery platform. I have two comments:
- The data in Figure 5 could be explained better with further supportive data. Specifically, it is unclear in panels 5e to h, how many mice were evaluated, and what 4-7 weeks or 16-24 weeks means. Please provide more detailed information of exactly when the serum was taken be provided, and additional show individual mouse biological replicates? Additionally, the investigation of single sex versus mixed sex is important, but it is unclear how rigorously this was set up to allow conclusions on whether there were any significant benefits of one sex choice compared to the other. It is also unclear why different numbers of worms were provided (40m versus 90f). More detail is needed, and information on the infection outcomes other than neutralizing potential of the serum could be provided, such as pathologic outcomes (weight, liver enzymes, tissue pathology after euthanasia).
- Figure 1 and the graphical summary abstract appear redundant. Figure 1 may be the better choice for the graphical abstract, and does not need to be included as a main figure.

Open Access This Peer Review File is licensed under a Creative Commons Attribution 4.0 International License, which permits use, sharing, adaptation, distribution and reproduction in any medium or format, as long as you give appropriate credit to the original author(s) and the source, provide a link to the Creative Commons license, and indicate if changes were made.
In cases where reviewers are anonymous, credit should be given to 'Anonymous Referee' and the source.
The images or other third party material in this Peer Review File are included in the article’s Creative Commons license, unless indicated otherwise in a credit line to the material. If material is not included in the article’s Creative Commons license and your intended use is not permitted by statutory regulation or exceeds the permitted use, you will need to obtain permission directly from the copyright holder.
To view a copy of this license, visit https://creativecommons.org/licenses/by/4.0/

2026-July-03

Responses to reviewers

Authors' point-by-point responses in blue font are provided below:

REVIEWERS' COMMENTS

Reviewer #1 (Remarks to the Author):

This manuscript by Ittiprasert reports some groundbreaking work in functional helminth genomics. The field of helminthology has long been plagued by the difficulty of successful and effective transgenics. While progress has been made in nematodes, this generation and propagation of a heritable transgenic schistosome line is certainly a breakthrough.

Additionally, the authors have utilized this approach of a transgenic parasite that lives in the blood to be an in vivo "living foundry" to continuously secrete a therapeutic neutralizing antibody within a mammalian host directly into the blood system. It is truly remarkable that they managed to get this to work. The manuscript is also well-written, the experimental data and design appear solid, and this work will likely have a high impact on the field.

Some of the strengths of their strategy is to deploy multiplexed CRISPR/Cas9 homology-directed knock-in targeting a predicted genome safe harbor (GSH1) in immature eggs. Propagating this line through snail and mouse hosts up to the F2 generation—where 97% of snails shed transgenic cercariae—is a remarkable technical achievement.

Interestingly, the authors decided to make the parasites express transgenic antibodies by fusing the anti-SARS-CoV-2 C5 VHH to a human IgG1 Fc domain. Driving expression with an endogenous, ubiquitously expressed schistosome ubiquitin promoter led to constitutive production without epigenetic silencing. They could demonstrate the functional utility of these transgenic anitbodies to neutralize live SARS-CoV-2.

My only critique would be that the authors need to have a more nuanced perspective regarding clinical safety and translation.

While Male-Only Infections have been used in controlled human infections, study participants still reported signs of Katayama fever, which acts earlier through an independent mechanism from the eggs that cause pathology in the liver. This systemic

hypersensitivity reaction to migrating schistosomula and maturing adults can cause significant morbidity, including fever, fatigue, myalgia, and eosinophilia. Hence the authors need to discuss the pathological risks of Katayama fever associated with male-only infections in the Discussion. Acknowledging this downside will provide a more balanced, comprehensive, and realistic view of the clinical hurdles facing the deployment of schistosomes as living foundries in humans.

We thank the reviewer for these comments. In response to the recommendation that "Acknowledging this downside will provide a more balanced, comprehensive, and realistic view of the clinical hurdles facing the deployment of schistosomes as living foundries in humans", we revised the Discussion to now include this additional sentence (penultimate paragraph), "Although male-only schistosome infections may induce symptoms of acute schistosomiasis when applied in high doses, repeated infections are extremely well tolerated, in line with the natural immunoregulatory nature of helminths[69]."

Reviewer #2 (Remarks to the Author):

The manuscript describes generation of stable transgenic lines of Schistosoma using CRISPR/Cas9 knock-in, and demonstrates that expression of an engineered antibody targeting the spike protein of SARS-CoV-2 from such transgene results in a functional antibody against SARS-CoV-2. This is a very significant technical advance, since stable and heritable transgenesis in Schistosoma will be instrumental for further investigations of this parasitic flatworm but also because it opens a venue for using this animal as a vehicle for delivery of therapeutic proteins.

The work is clearly described and technically solid. I'd like to congratulate the authors!

We appreciate the reviewer's description of the paper and congratulations.

I have only several comments:
Lines 67-68: "To date, heritably transgenic lines of schistosomes or indeed any platyhelminth –parasitic or free living - have not been described." – This is not correct and disappointing that the authors are not sufficiently informed about broader platyhelminth literature. Stable transgenesis in a free-living flatworm Macrostomum was described in 2017 (also in Nature Communications), and several follow-up papers with more transgenic lines have been published since.

Aiming to revise our manuscript to address the reviewer #2's concern about lack of acknowledgement of earlier reports on transgenesis in the Platyhelminthes, the opening sentence of Introduction has been revised to:
"To date, heritably transgenic lines of schistosomes, or of any parasitic platyhelminth (clade Neodermata within phylum Platyhelminthes), have not been described". In addition, we revised the Discussion as follows,

"Notwithstanding our success with schistosomes with these methods, however, transgenesis approaches from the planarian *Macrostomum lignano* also may be useful in parasitic flatworms [55,56]. "

Lines 258-260 – primer sequences not needed here but should be in the table with all primers.

All primers are reported now only in Supplementary Table 1.

The efficiency of CRISPR/Cas9 knockin is not discussed. The numbers are mentioned in the text but a small table will be very useful. If I understand correctly, the numbers are based on a single experiment. Do the authors have more information about the reproducibility of knock-ins?

Some very specific choices were made in the knock-in protocol, e.g. the use of oligos modified in a certain way. A discussion about these particular choices and potential alternatives that might further increase the efficiency will be useful.

We have included the following paragraph in the Discussion to address this request and our particular choices and potential alternatives:

"Reproducibility of targeted knock-ins can be variable across systems[62,63]. Although we report the findings of one primary electroporation experiment, prior work from our group and others indicates consistent outcomes: IVLE electroporation with overlapping Cas9 RNPs and modified dsDNA donors typically yields overall editing (indel+HDR) of ~20–30% and reporter-positive rates among edited eggs of ~60–80% when electroporation is optimized (e.g., 125 V); higher voltages reduce hatch rates[12]. The lifecycle of *S. mansoni* (egg→egg) is ~ 3 months under favorable laboratory conditions, and it took 9–12 months from construct design for the C5-Fc transgene to obtain the F2 generation in this study, a constraint on performing many independent replicates. Finally, retroviral transduction of IVLEs (versus liver eggs) produces substantially higher transgene copy numbers in downstream cercariae and reliable germline transmission, albeit with random integration[16]."

Aiming to address Reviewer #4's request, and the related request from the Editor, "More detail is needed, and information on the infection outcomes other that neutralizing potential of the serum, such as pathologic outcomes (weight, liver enzymes, tissue pathology, euthanasia".

To address the Editor's related request,

"Please be advised that adding additional pathological outcomes of your proof-of-concept antibody delivery part, as suggested by reviewer #4 would be appreciated if readily available.", as well as the specific comment from reviewer #4,
"...and information on the infection outcomes other than neutralizing potential of the serum could be provided, such as pathologic outcomes (weight, liver enzymes, tissue pathology after euthanasia)."

We have included additional findings (which were not included in the original submission) in the Supplementary Information, specifically Supplementary Figure 4, panels c and d and Supplementary Figure 5. In addition, we have included two short movies, Supplementary Videos 1 and 2, and revised the Results and Discussion to present and discuss the additional information. The new text included in the R1 version is as follows:

"**Results**

Stability and maintenance of C5-Fc transgenic line

Finally, in addition to detailed information collected during the establishment of the transgenic line (above), the C5-Fc line was subsequently maintained by routine laboratory passage: naïve snails exposed to 6–10 miracidia and mice to 100–200 cercariae. Periodic sampling of genomic DNAs and total RNAs of adult stages during generations F3 to F9 confirmed transmission of the transgene while, concurrently, hatching of eggs recovered from mouse livers, motility and infectivity of miracidia and cercariae, presence of paired adult worms in the portal circulation at mouse necropsy, and hepatosplenomegaly and hepatic granulomas of host mice, confirmed the viability and, vitality of the transgenic schistosome line. Supplementary Figures 5, 6 and Supplementary Movies 1, 2 provide representative gels and images representative of these findings among these later C5-Fc generations, including the salient finding by western blot that F8 generation transgenic schistosomes secreted the C5-Fc antibody into the circulation of the host mice.

"**Discussion**

Parental (female and male) P0 transgenic S. mansoni were established and used to infect mice; eggs from these mice produced the F1 generation, and miracidia from F1 eggs infected snails to propagate the C5-Fc line into the F2 generation (Figure 4). The line was maintained for multiple subsequent generations while retaining the full life cycle, infectivity, and virulence of the parental NMRI strain, demonstrating a robust, tractable platform for molecular and therapeutic studies. Importantly, western blotting shows F8 parasites continue to secrete the C5-Fc antibody into the host bloodstream (Supplementary Figure 5), validating the GSH1 site on chromosome 3 as a reliable locus for future transgenic lines and establishing an entirely novel biological drug-delivery platform."

"Supplementary Figure 5. Representative observations of C5-Fc transgenic *Schistosoma mansoni* across generations through F9.

Panel **a.** Photographs of mouse livers resected at necropsy 62 days after infection with 105 cercariae of the F6 generation of the transgenic line, showing hepatic granuloma. **b.** PCR confirmation of C5-Fc transgene in genomic DNA from F5-generation transgenic C5-Fc schistosomes. Representative agarose gel stained with SYBR SAFE showing PCR products generated with transgene-specific primers C5VHH-F and FcIgG1-R. Lanes: 1, DNA size marker; 2, primers only (no template control); 3, C5-Fc F5 gene genomic DNA (gDNA), no primers; 4, wild-type (negative control) genomic DNA from the NMRI strain of *S. mansoni* (no band at expected transgene size); 5–6, gDNA from two independent pools of F5-generation transgenic adult, mixed sex *S. mansoni* worms, showing the expected C5-Fc amplicon of 302 nt (blue arrow); 7, empty lane; 8,. Positive control – primers, 70 pg donor transgene plasmid (positive control), showing the expected C5-Fc amplicon of 302 nt (blue arrow). PCR conditions: [primers C5VHH-F and FcIgG1-R], annealing temperature 55 °C, 35 cycles. Gel: 1% agarose, 96 volts for 30 minutes. The presence of the 302 bp C5-Fc amplicon in two independent F5 transgenic gDNA pools (lanes 5–6) and the plasmid positive control, with no band in wild-type or no-template controls, confirmed stable genomic integration of the C5-Fc transgene in the F5 generation. **c.** Pairs of F6 generation adult worms *in copula,* perfused from the portal circulation of mice. Length of worms, ~ 10 mm each. **d.** End-point RT-PCR detection of C5-Fc transgene and GAPDH in adult *Schistosoma mansoni* cDNA. Lane 1, SYBR SAFE–stained 1% agarose gel of RT-PCR products run alongside a 1 kb Plus DNA ladder (Invitrogen); 2, total cDNA/RNA from adult *S. mansoni* and transgene primers C5VHH-F and FcIgG1-R, with a clear band visible at 302 bp corresponding to the C5-Fc transgene;

3, primers without cDNA; 4, lane is empty; 5, total cDNA/RNA from adult *S. mansoni* and *Smgapdh* primers C5VHH-F and FcIgG1-R, with a clear band visible at 302 bp corresponding to the C5-Fc transgene, and the strong band at 285 bp corresponded to the reference gene *Smgapdh*. 6, 1 kb Plus DNA ladder; 7 lane is empty; 8, positive control, 70 pg donor transgene plasmid (positive control), transgene primers, and displays the expected 302 bp band. Bands were visualized with SYBR SAFE. Amplificon of the 302 bp fragment of the C5-Fc transgene in adult *S. mansoni* cDNA with robust *Sm*GAPDH (285 bp) and the absence of any product in the no-template control confirmed specific transgene transcription, validated by the plasmid positive control. **e.** Photographs documenting hatching of C5-Fc F9 generation eggs and swimming of miracidia in water in a beaker (left and right sides show low and higher magnifications, respectively. **f.** Western blot of proteins separated on a 4–1% gradient SDS–PAGE and probed with HRP-conjugated rabbit IgG monoclonal anti–6×His tag (1:5,000). Lane 1: prestained protein standards (Sea Blue Plus2, Fisher Scientific). Lanes 2–4: individual mouse sera (1.5 µl per lane) from three separate mice infected with F8-generation C5–Fc transgenic *S. mansoni* (one mouse per lane), sampled at day 70 after infection with 135 cercariae. Lanes 5–7: individual mouse sera (1.5 µL per lane) sampled at day 50 after infection with.180 cercariae of wild-type *S. mansoni* (one mouse per lane). Lanes 8–10: recombinant C5–Fc produced in HEK-293 cells (2 ng in lane 8; 10 ng in lane 9 and 20 ng in lane 10). Molecular mass markers indicated at left; expected position of C5–Fc is marked with a blue arrow in the C5-Fc mouse sera and black arrow in recombinant C5-Fc lanes. The presence of the C5–Fc signal in mouse sera confirmed continued inheritance and functional expression of the C5–Fc transgene through at least eight filial generations. The PCR based assays shown in panels b and d were repeated twice, with similar results both times, and the western in panel f was repeated three times, with sera from the same mice, with the same outcome for each replicate.

Supplementary Movie 1. Hatching of eggs and swimming *Schistosoma mansoni* miracidia in pond water. The eggs and miracidia are from the F9 generation of the of the C5-Fc line. Video recorded on an iPhone 16 showing a beaker containing pond water with schistosome eggs recovered from the livers of mice experimentally infected with F8 generation C5-Fc schistosomes. Miracidia are hatching from the eggs, which are on the bottom of the beaker, and swim actively in the water. Playback speed is real time (1×); scale bar ~200 µm (estimated using egg length ~120 µm). Brightfield illumination; no staining. File format: MP4; duration 00:00:25; resolution 1920×1080.

Supplementary Movie 2. *Biomphalaria glabrata* snail during exposure to infection with miracidia of F9 generation *Schistosoma mansoni*. Continuous video recorded on an iPhone 16 showing a single *B. glabrata* adult (shell diameter ≈ 5 mm) following the

addition of recently hatched *S. mansoni* miracidia to the observation field; ~100 miracidia dispensed into a beaker holding 10 snails in 10 ml pond water . Following the miracidia addition, snails exhibited movements tentacles and partial withdrawal into the shell, episodic body extension and locomotory changes,. Scale bar = 5 mm (shell diameter reference). Lighting and focus reflect handheld smartphone video capture in standard laboratory lighting. "

Reviewer #3 (Remarks to the Author):

Summary

The authors of this paper demonstrate 1) stable introduction of a new gene that is now heritable into a platyhelminth, and
2) ability to utilize schistosomes as vehicles to produce novel proteins for long periods of time in an infected host. The authors used CRISPR/Cas9 technology to knock-in a transgene construct "C5-Fc" encoding an anti-SARS-COV-2 spike VHH fused to the human IgG1 Fc domain. They utilized three overlapping guide RNAs for the CRISPR/Cas9 knock-in and included the S. mansoni ubiquitin promoter and a secretion signal peptide from the hookworm protein ASP2. The resultant C5 Fcs dimerized, producing a bivalent antibody against SARS-CoV-2. Transfection was conducted via electroporation of the three guide RNAs along with the C5-Fc gene into immature schistosome eggs. Miracidia hatched from these eggs were then used to infect snails, and then cercariae from the snail used to infect mice. Mice were infected sequentially with male cercariae and then, a few weeks later, with female cercariae. Eggs obtained from the murine infection were then used to generate F1 generation miracidia to infect snails, from which cercariae were derived to infect a 2nd generation of mice. The authors show that both excretory/secretory product from transgenic flukes, as well as sera from infected mice, have C5-Fc (by ELISA) which can effectively neutralize SARS-CoV-2 (shown via both lentiviral pseudovirus assay as well as plaque reduction assay using live virus.

Overall review

Overall, the paper is quite remarkable, as it is the first to my knowledge to demonstrate stable introduction of a new, heritable gene into a platyhelminth. Additionally, the utilization of Schistosomes as a potential long-lived factory for production of a specific protein in an infected host is novel and may have important translational implications (if not with Schistosomes, then perhaps with a less pathogenic long-lived helminth).
The paper is well written, the scientific approach appears robust, and the figures, especially the one on assembly of the transgene, are very clear and easy to follow. The methods are also exceptionally well written, with sufficient detail to enable replication of the study.

Major critiques: none

Minor critiques:

1) In Figure 5, when using sera of infected mice, the authors should specify whether the sera was from mice infected with the first round of cercariae (F1) or the 2nd round (F2).

These sera were from mice exposed to the P0 or the F1 generation of the C5-Fc transgenic line.

We have revised the legend of Figure 5 to include these details,

"Sera in panels e, f, g and h were from mice exposed to the P0 or the F1 generation of the C5-Fc transgenic line".

2) If the data from the serum studies in Figure 5 is from the F1 generation, did the authors conduct studies to confirm that the F2 continued to produce C5-Fc in vivo? This may have been mentioned somewhere in the text but I didn't catch it.

Whereas we did not examine whether the F2 generation produced C5-Fc in vivo, we now show that subsequent filial generations of the transgenic schistosomes did indeed continue secreting the C5-Fc antibody into the circulation of infected mice. To illustrate this salient finding, we included a western blot in Supplementary Figure 5 (panel F). This blot confirmed the presence of the C5-Fc antibody in blood in three of three mice infected with the F8 generation of the transgenic line. In addition, panels b and d confirmed the presence and expression of the transgene in generations later than F2. This issue is now addressed in the Discussion and Supporting Information (see above).

Reviewer #4 (Remarks to the Author):

This is a remarkable study demonstrating the generation of transgenic schistosoma lines that secrete neutralizing nanobodies to SARS-CoV2. Together, the authors combine CRISPR/Cas, nanobody design, and Schistosoma manipulation for a molecular tour-de-force in generating heritable parasites that secrete a functional therapeutic protein against SARS-CoV2. Prior studies have reported the generation of transgenic helminths, but this work is the first of its kind in generating heritably transgenic schistosoma lines that produce a therapeutic protein. Based on this novelty, this study will be of high significance to many fields of research, from therapeutic technologies to parasitology and immunology.

The study provides thorough detail and rational for the design and validation of the transgenic parasites, and reports several findings: 1/ the successful generation of transgenic schistosoma that produce the camelid antibody against SARS-CoV2, specifically VHH-C5-Fc that was the most potent in plaque assays, 2/ the successful infection of mice with single or mixed sexes that resulted in long-term secretion (24 weeks) of the functional nanobody in the mouse blood, 3/the strong neutralization potential for the nanobodies from the infected mouse serum.
Overall, this is a well-written study with convincing results and provides the research field with a new therapeutic delivery platform. I have two comments:

1. The data in Figure 5 could be explained better with further supportive data. Specifically, it is unclear in panels 5e to h, how many mice were evaluated, and what 4-7 weeks or 16-24 weeks means. Please provide more detailed information of exactly when the serum was taken be provided, and additional show individual mouse biological replicates?

Q: what 4-7 weeks or 16-24 weeks means?

Figure 5 legend has been expanded to explain the serum collection time points: "C5-Fc secreted by transgenic worms into mouse blood and serum collected 4-24 wk pos-infection. "

Q: how many mice were evaluated

Biological replicate mouse n values have been added to the legend of each panel.

Additionally, the investigation of single sex versus mixed sex is important, but it is unclear how rigorously this was set up to allow conclusions on whether there were any significant benefits of one sex choice compared to the other.

Both male and female single sex schistosomes have been used in controlled human infection studies: PMID: 32066978. and PMID: 39666392. However, given our long-term goal of using transgenic schistosomes in vivo as a drug delivery platform, using male rather than female single-sex infections may be considered safer, because the pathology is driven by eggs released by the female parasite.

It is also unclear why different numbers of worms were provided (40m versus 90f).

At that stage in the transgenic line derivation, the number of cercariae shed from individual snails (each infected with a single miracidium) was finite. Therefore, the size of the clonal populations of single sex cercariae available on the day mice were infected defined the number of cercariae available for mouse infection. Hence, the data we

collected were determined by this biological constraint. Notwithstanding, our findings showed that both female and male schistosomes released the C5-Fc antibody into the blood of infected mice.

More detail is needed, and information on the infection outcomes other than neutralizing potential of the serum could be provided, such as pathologic outcomes (weight, liver enzymes, tissue pathology after euthanasia).

We addressed this issue in the R1 version. As noted above (pages 4, 5), we have detailed the changes made in response to the Editor's request: "Please be advised that adding additional pathological outcomes of your proof-of-concept antibody delivery part, as suggested by reviewer #4 would be appreciated if readily available," as well as the specific comment from reviewer #4: "...and information on the infection outcomes other than neutralizing potential of the serum could be provided, such as pathologic outcomes (weight, liver enzymes, tissue pathology after euthanasia)."

2. Figure 1 and the graphical summary abstract appear redundant. Figure 1 may be the better choice for the graphical abstract and does not need to be included as a main figure.

The graphic abstract has been removed.
