# Supplementary material for: Heritable transgenic schistosomes as a living platform for SARS-CoV-2 neutralizing antibody secretion
Source: Nat Commun. 2026 Aug 26;17:9052. doi: 10.1038/s41467-026-76465-9 (PMC13518888; doi:10.1038/s41467-026-76465-9)
Supplement: Supplementary file 4 [file 41467_2026_76465_MOESM2_ESM.docx]

**Description of Additional Supplementary Files**

**Supplementary Movie 1.**

Hatching of Schistosoma mansoni eggs of F9 generation 269 of the C5-Fc line, with the released miracidia actively in a beaker of pond water.

**Supplementary Movie 2.**

Biomphalaria glabrata snail during exposure to infection 278 with miracidia of the F9 generation of transgenic S. mansoni.
